# Solar light induced photocatalytic degradation of tetracycline in the presence of ZnO/NiFe_2_O_4_/Co_3_O_4_ as a new and highly efficient magnetically separable photocatalyst

**DOI:** 10.3389/fchem.2022.1013349

**Published:** 2022-10-13

**Authors:** Mohammadreza Doosti, Roya Jahanshahi, Shaghayegh Laleh, Sara Sobhani, José Miguel Sansano

**Affiliations:** ^1^ Department of Civil Engineering, Faculty of Engineering, University of Birjand, Birjand, Iran; ^2^ Department of Chemistry, College of Sciences, University of Birjand, Birjand, Iran; ^3^ Departamento de Química Orgánica, Facultad de Ciencias, Centro de Innovación en Química Avanzada (ORFEO-CINQA) and Instituto de Síntesis Orgánica (ISO), Universidad de Alicante, Alicante, Spain

**Keywords:** wastewater treatment, solar light, photocatalysis, heterogeneous catalysis, magnetically separable, tetracycline, degradation

## Abstract

In this study, a new solar light-driven magnetic heterogeneous photocatalyst, denoted as ZnO/NiFe_2_O_4_/Co_3_O_4_, is successfully prepared. FT-IR, XPS, XRD, VSM, DRS, FESEM, TEM, EDS, elemental mapping, and ICP analysis are accomplished for full characterization of this catalyst. FESEM and TEM analyses of the photocatalyt clearly affirm the formation of a hexagonal structure of ZnO (25–40 nm) and the cubic structure of NiFe_2_O_4_ and Co_3_O_4_ (10–25 nm). Furthermore, the HRTEM images of the photocatalyst verify some key lattice fringes related to the photocatalyt structure. These data are in very good agreement with XRD analysis results. According to the ICP analysis, the molar ratio of ZnO/NiFe_2_O_4_/Co_3_O_4_ composite is obtained to be 1:0.75:0.5. Moreover, magnetization measurements reveals that the ZnO/NiFe_2_O_4_/Co_3_O_4_ has a superparamagnetic behavior with saturation magnetization of 32.38 emu/g. UV-vis DRS analysis indicates that the photocatalyst has a boosted and strong light response. ZnO/NiFe_2_O_4_/Co_3_O_4_, with band gap energy of about 2.65 eV [estimated according to the Tauc plot of (αhν)^2^ *vs*. hν], exhibits strong potential towards the efficacious degradation of tetracycline (TC) by natural solar light. It is supposed that the synergistic optical effects between ZnO, NiFe_2_O_4_, and Co_3_O_4_ species is responsible for the increased photocatalytic performance of this photocatalyst under the optimal conditions (photocatalyst dosage = 0.02 g L^−1^, TC concentration = 30 mg L^−1^, pH = 9, irradiation time = 20 min, and TC degradation efficiency = 98%). The kinetic study of this degradation process is evaluated and it is well-matched with the pseudo-first-order kinetics. Based on the radical quenching tests, it can be perceived that ^•^O_2_^−^ species and holes are the major contributors in such a process, whereas the ^•^OH radicals identify to have no major participation. The application of this methodology is implemented in a facile and low-cost photocatalytic approach to easily degrade TC by using a very low amount of the photocatalyst under natural sunlight source in an air atmosphere. The convenient magnetic isolation and reuse of the photocatalyst, and almost complete mineralization of TC (based on TOC analysis), are surveyed too, which further highlights the operational application of the current method. Notably, this method has the preferred performance among the very few methods reported for the photocatalytic degradation of TC under natural sunlight. It is assumed that the achievements of this photocatalytic method have opened an avenue for sustainable environmental remediation of a broad range of contaminants.

## 1 Introduction

Over the recent decades, the utilization of solar energy as a renewable, sustainable, clean, abundant, and cheap alternative for the remediation of contaminated wastewater has attracted tremendous attention (Yang et al., 2019; Lim et al., 2020). Following this line, photocatalysis techniques that are associated with using heterogeneous semiconductors, as one of the most well-known advanced oxidation processes (AOPs), have been found to be a promising strategy (Loddo et al., 2018). Heterogeneous semiconductor photocatalysis is an advantageous approach with cost-effectiveness, sustainability, versatility, and environmental compatibility, which could effectually increase the mineralization of the pollutants (Dai et al., 2021; Khan and Yadav, 2021). However, a habitual obstacle facing the photocatalytic performance is having a large band gap with a low capability of using solar energy and the speed of the generated electron/hole pairs recombination, rendering them inappropriate for practical uses (Ma et al., 2014). To address these shortcomings, the development of more efficient heterostructured nanocomposite photocatalysts composed of two or more active components is associated with the reduction in band gap values and prolonged the lifetime of the separated electron and holes careers (Shan et al., 2017; Shan et al., 2022), which improve their practical response under the solar energy (Lang et al., 2014; Dong et al., 2015).

Zinc oxide (ZnO) is one of the most well-known semiconductors, which has been widely used as an excellent photocatalyst (Saravanan et al., 2013). Due to its desirable properties, including the superb optical and electrical attributes, non-toxicity, chemical stability, and low cost, it has been considered as a unique candidate for photocatalytic wastewater treatments (Weldegebrieal, 2020). Nevertheless, the wide band gap energy (Eg = 3.37 eV) of ZnO makes it to absorb only a small portion of sunlight (UV light) (Tan et al., 2016). This fact severely limits its applications under visible light. Therefore, there is a continued interest to improve the photocatalytic efficiency of ZnO-based photocatalysts towards the visible and/or sunlight irradiation for practical usage. In this regard, one of the most operational methodologies is the combination of ZnO with qualified metal oxide semiconductors to prepare composite photocatalysts (Ramírez et al., 2021).

Nickel ferrite (NiFe_2_O_4_) is a promising magnetic, non-toxic, cost-effective, and thermal/chemical resistant material with a narrow band gap energy (Eg = 1.7 eV) (Ren et al., 2014; Rana et al., 2021). It has been found to be a perfect metal oxide semiconductor for numerous technological and environmental applications (Sharifi et al., 2012; Abu-Dief et al., 2016; Lasheras et al., 2016; Zhang et al., 2018; Umut et al., 2019; Reddy et al., 2020; Shinde et al., 2021). Fascinatingly, the incorporation of NiFe_2_O_4_ into the ZnO-based photocatalytic systems effectually improves the visible light absorption potential of the photocatalyst by enhancing the ability to separate the photo-excited electron/hole pairs (Rahmayeni et al., 2016; Zhu et al., 2016; Chandel et al., 2020). Furthermore, the magnetic behavior of NiFe_2_O_4_ facilitates the separation and recycling process of the photocatalyst by using an external magnetic field.

In recent years, cobalt oxide (Co_3_O_4_) has received extensive scientific attention in the field of photocatalysis due to having excellent electronic properties, low solubility, high thermal and chemical stability, and strong visible light absorption (Han et al., 2014; Zhang et al., 2016; Shao et al., 2017; Wu et al., 2018). In general, the addition of Co_3_O_4_ semiconductor with a narrow band gap energy of 2.07 eV to the photocatalytic system synergistically provides excellent stability and effectively raises the ability of electrons and holes to separate and so leads to better photocatalytic performance under solar light irradiation (Liu et al., 2017).

Based on these premises and to pursue the intended endeavors of our research groups towards the development of new photocatalytic systems operating in mild conditions (Jahanshahi et al., 2020a; Jahanshahi et al., 2020b), herein, for the first time, we synthesized a ZnO/NiFe_2_O_4_/Co_3_O_4_ nanocomposite as a highly efficient magnetically separable heterogeneous sunlight-driven photocatalyst. The as-synthesized photocatalyst was entirely characterized by various methods. The ability of ZnO/NiFe_2_O_4_/Co_3_O_4_ was evaluated towards the photocatalytic degradation of tetracycline (TC) under natural sunlight irradiation.

## 2 Materials and methods

### 2.1 Chemicals and reagents

All chemicals and solvents used in our experiments were purchased from Sigma-Aldrich and Merck chemical companies and were used directly without further purification. TC tablets (250 mg) were provided by Tolid Darou pharmaceutic company. Throughout the study, deionized water was used wherever needed. For pH adjustment, hydrochloric acid and sodium hydroxide were utilized.

### 2.2 Instrumentation

The progress of the degradation process was monitored by UV-vis spectrophotometer (Shimadzu, 2501-PC, Kyoto, Japan). FT-IR spectra were recorded with a JASCO FT-IR 460 plus spectrophotometer within the 400–4000 cm^−1^ range using KBr disc at room temperature. XRD was carried out an Xpert Pro Panalytical diffractometer (PW1730, PHILIPS company) with Cu Kα radiation (*λ* = 1.540 Å). VSM analysis was done using Magnetic Daghigh Kavir apparatus, as a homemade instrument (MDKB model, Iran). XPS measurements were performed using a VG-Microtech Multilab 3000 spectrometer, equipped with an Al anode, and the deconvolution of related XPS spectra was accomplished by Gaussian Lorentzian curves. TEM images were obtained using a TEM microscope JEOL JEM-1400 Plus device. FESEM microscopy is performed in a Hitachi model S3000N. UV-vis DRS of samples was obtained using a Shimadzu spectrophotometer (UV-2550 model). EDS and elemental mapping are conducted on a TESCAN MIRA3 instrument. The content of elements in the photocatalyst was determined with an OPTIMA 7300DV ICP analyzer. Total organic carbon (TOC) was measured using the Shimadzu TOC-VCSN analyzer. A glass-combination of electrode equipped with digital pH-meter (HANNA instruments HI 2211 pH/ORP Meter) was utilized for the pH control.

### 2.3 Fabrication of the photocatalyst

#### 2.3.1 Synthesis of NiFe_2_O_4_ NPs

Ni(NO_3_)_2_.6H_2_O (20 mmol) and Fe(NO_3_)_3_.9H_2_O (20 mmol) were added to the deionized water (100 ml) and stirred vigorously on a magnetic stirrer for 30 min. Thereafter, NaOH solution (2 M) was added dropwise into the mixture under constant stirring until the pH value was adjusted at 12. The obtained solution was heated at 180°C in a Teflon-lined stainless autoclave for 3 h. Next, the autoclave was allowed to cool at ambient temperature and the resultant sample was collected and washed repeatedly with distilled water. After the suspension reached a pH of 7, the obtained product was vacuum-dried at 70°C for 2 h to afford the desired NiFe_2_O_4_ nanoparticles (Rahmayeni et al., 2016).

#### 2.3.2 Synthesis of ZnO/NiFe_2_O_4_ NPs

ZnO/NiFe_2_O_4_ nanoparticles were obtained by the simple solvothermal method. The previously synthesized NiFe_2_O_4_ nanoparticles (0.02 g) were mixed with Zn(NO_3_)_2_.4H_2_O (0.05 g) and the resulting mixture was dissolved in ethanol (40 ml) and then mixed with magnetic stirring within 30 min. Then, to adjust the pH value at 12, NaOH was added dropwise to the solution under vigorous stirring. The resulting sample was annealed at 180°C for 3 h in a Teflon-lined stainless autoclave. After cooling down to room temperature, the obtained product was washed repeatedly with distilled water until the solution was neutralized. ZnO/NiFe_2_O_4_ NPs were finally attained after the vacuum-drying of the as-synthesized products at 70°C for 2 h (Rahmayeni et al., 2016)

#### 2.3.3 Synthesis of ZnO/NiFe_2_O_4_/Co_3_O_4_

ZnO/NiFe_2_O_4_ NPs (1.0 g) was dispersed in 100 ml of an H_2_O: EtOH mixture (1:1, v/v) under ultrasonication for 30 min. Subsequently, Co(NO_3_)_2_.6H_2_O (1.5 g, 5.19 mmol) was mixed with the resulting suspension and stirred continuously for 1 h. The obtained product was dried at 70°C in a vacuum oven for 3 h, before being annealed at 300°C (heating rate of 5°C min^−1^) for 2 h (Mousavi and Habibi-Yangjeh, 2017). According to the ICP analysis, the molar ratio of ZnO/NiFe_2_O_4_/Co_3_O_4_ composite was obtained to be 1:0.75:0.5.

### 2.4 Photocatalytic degradation experiments

Typically, the photocatalytic capability of ZnO/NiFe_2_O_4_/Co_3_O_4_ was evaluated towards the degradation of TC under solar light irradiation. In all experiments, a batch reactor (500 ml) was used as the reaction vessel and the reaction temperature was tried to maintain at 25°C ± 3°C by keeping the reaction vessel in a water bath. The degradation performance of ZnO/NiFe_2_O_4_/Co_3_O_4_ was tested by studying various parameters comprising the pH value (3–11), catalyst quantity (0.005–0.05 g L^−1^), TC concentration (10–30 mg L^−1^) and contact time (5–60 min). Before irradiation, the reaction suspension was stirred in the dark for 30 min to ensure the equilibrium of adsorption and desorption. For every run of the degradation process, oxygen blowing into the suspension was ensured using an air pump. The reaction was conducted under a continuous magnetic stirring, which can guarantee the homogeneity of the solution. The solution was exposed to natural sunlight irradiation from 11:00 a.m. to 3:00 p.m. on summer days. At certain time intervals (5 min) of the photocatalytic degradation process, 3 ml portions of the solution were collected from the reaction solution, followed by magnetic isolation of the catalyst. The concentration of the remaining TC was measured at its λ_max_ (357 nm) using a UV−vis spectrophotometer. The following equation has been used to evaluate the photocatalytic efficiency of the TC degradation process:

$$\mathrm{Degradation}\;e\;f\;f\mathrm{iciency}\;\left(D.E.\right)\left(\%\right)=\frac{C_{0}-C_{t}}{C_{0}}\times 100$$

where C_o_ is related to the initial concentration and C_t_ is related to the final concentration of TC after the irradiation time of t.

### 2.5 Photocatalytic degradation experiments in the presence of capture agents

To find the main reactive species in this process, the degradation of TC was studied in the presence of the free radical/hole trapping agents. These experiments were set up and conducted in the same way as a typical degradation experiment individually in the presence of isopropanol (IPA), benzoquinone (BQ), and ammonium oxalate (AO), which act in turn as the hydroxyl radical (^•^OH), superoxide radical (^•^O_2_^−^) and hole (h^+^) scavengers. In this line, the TC photocatalytic degradation efficiency was investigated in the presence of each scavenger (1 mM) under the optimized conditions (photocatalyst dosage = 0.02 g L^−1^, TC concentration = 30 mg L^−1^, pH = 9, and irradiation time = 20 min), and solar light irradiation. In all experiments, a 500 ml batch reactor was used as the reaction vessel and the reaction temperature was maintained at 25°C ± 3°C by keeping the reaction vessel in a water bath. Before irradiation, the reaction suspension was stirred in dark for 30 min to guarantee the adsorption-desorption equilibrium. For every run, oxygen blowing into the suspension was ensured using an air pump. The reaction was conducted under a continuous magnetic stirring, which can provide the homogeneity of the solution. The solution was exposed to natural sunlight irradiation from 11:00 a.m. to 3:00 p.m. on summer days. At certain time intervals (5 min) of the degradation process, 3 ml portions of the solution were collected from the reaction solution, followed by magnetic isolation of the catalyst. The concentration of the remaining TC was conventionally measured using a UV−vis spectrophotometer.

## 3 Results and discussion

### 3.1 Preparation and characterization of ZnO/NiFe_2_O_4_/Co_3_O_4_

**FIGURE 1:**
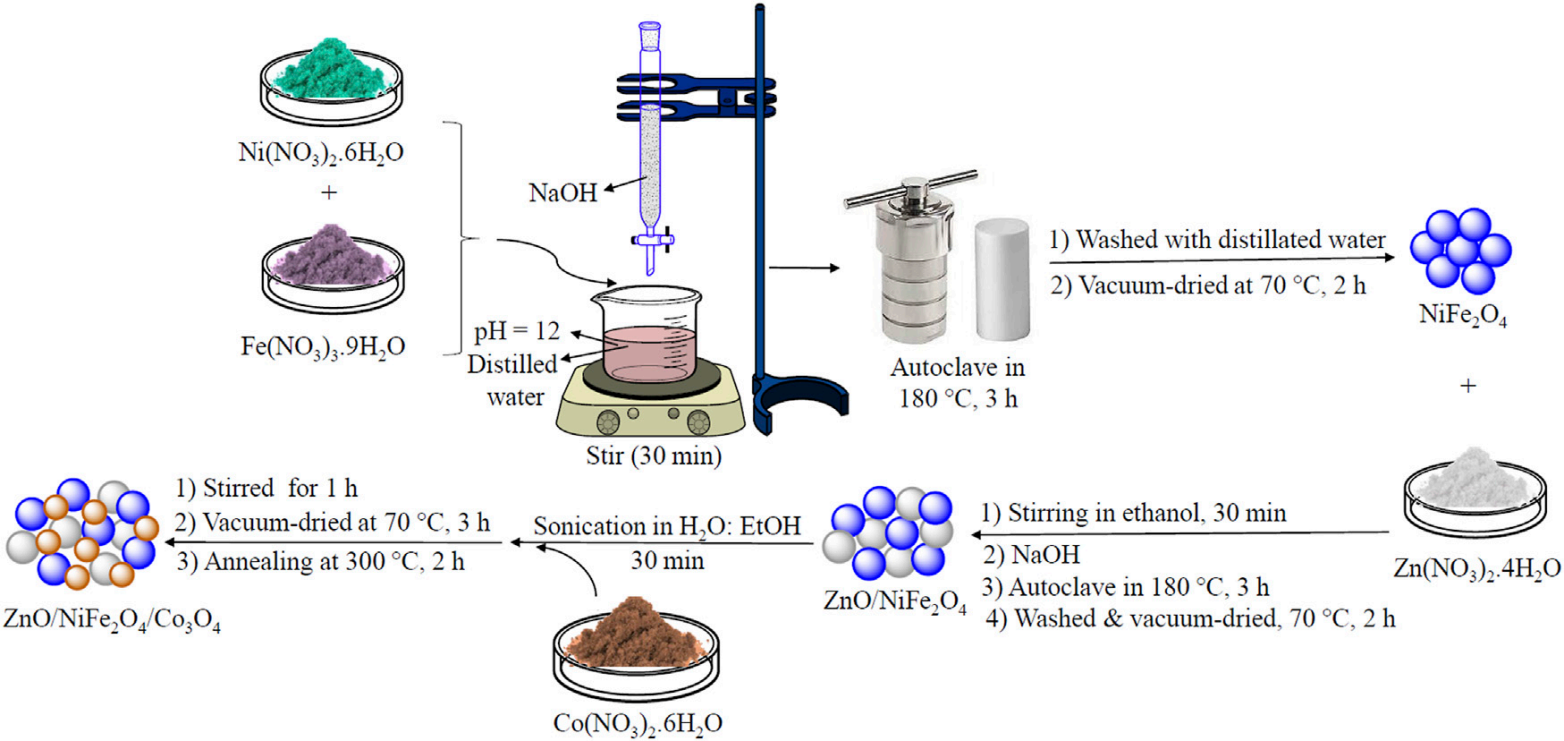
Schematic illustration of the fabrication process of the ZnO/NiFe_2_O_4_/Co_3_O_4_ nanocomposite.

As shown in Figure 1, ZnO/NiFe_2_O_4_/Co_3_O_4_ was synthesized according to the overall multi-step synthetic process as follows: firstly, NiFe_2_O_4_ NPs were prepared from a mixture of Ni(NO_3_)_2_·6H_2_O (20 mmol) and Fe(NO_3_)_3_.9H_2_O, using a typical hydrothermal procedure (Rahmayeni et al., 2016). Secondly, the fabricated NiFe_2_O_4_ NPs were treated with Zn(NO_3_)_2_·4H_2_O through a similar solvothermal approach to generate ZnO/NiFe_2_O_4_ NPs. In the last step, the addition of Co(NO_3_)_2_·6H_2_O to the H_2_O/EtOH solution of ZnO/NiFe_2_O_4_ under vigorous stirring, followed by the annealing process at 300°C, afforded the desired ZnO/NiFe_2_O_4_/Co_3_O_4_ NPs. The freshly synthesized ZnO/NiFe_2_O_4_/Co_3_O_4_ was fully characterized by different techniques.

#### 3.1.1 Fourier transform infrared analysis

**FIGURE 2:**
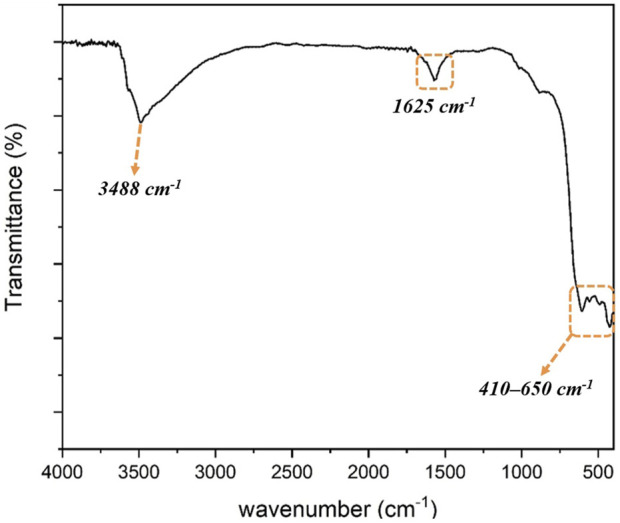
FT-IR spectrum of ZnO/NiFe_2_O_4_/Co_3_O_4_.

Fourier transform infrared (FT-IR) spectrum of ZnO/NiFe_2_O_4_/Co_3_O_4_ is shown in Figure 2. In general, the indicative absorption bands relating to the metal-oxygen bands stretching vibrations are presented at about 410–650 cm^−1^. To express in more detail, it can be considered that the absorption bands at 420, 493, 555, and 604 cm^−1^ could be ascribed to the stretching vibration frequencies of Fe-O, Zn-O, Ni-O, and Co-O bonds, respectively (Habibi and Parhizkar, 2014; Sharma et al., 2016; Naidu and Narayana, 2019; Velmurugan et al., 2021). Furthermore, an absorption band located at 1625 cm^−1^, and a broad band centered at 3488 cm^−1^, are respectively allocated to bending and stretching modes of the physically adsorbed H_2_O molecules on the catalyst surface.

#### 3.1.2 X-ray diffraction analysis

**FIGURE 3:**
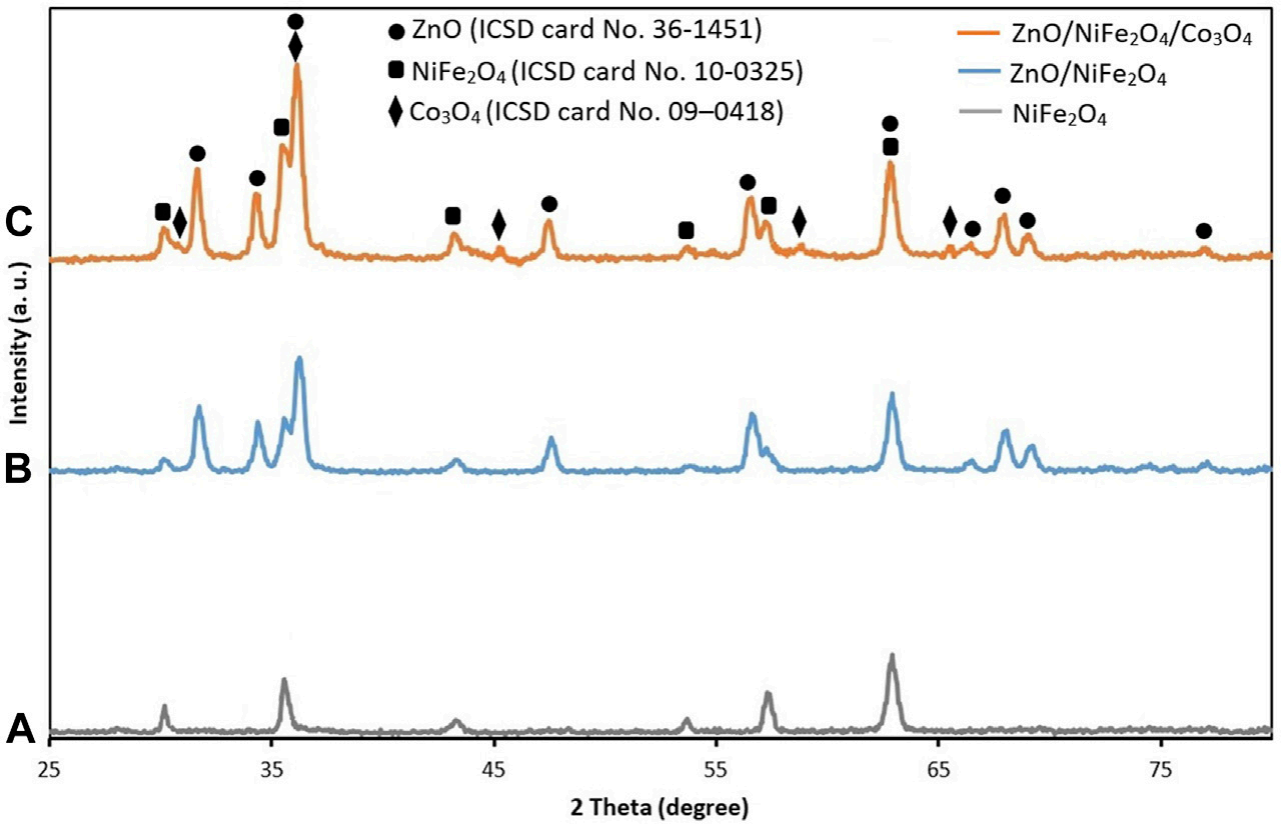
XRD patterns of **(A)** NiFe_2_O_4_, **(B)** ZnO/NiFe_2_O_4_ and **(C)** ZnO/NiFe_2_O_4_/Co_3_O_4_.

X-ray diffraction (XRD) analysis of NiFe_2_O_4_, ZnO/NiFe_2_O_4_ and ZnO/NiFe_2_O_4_/Co_3_O_4_ were implemented to emphasize the structural features of the catalyst (Figure 3). Signals positions with 2θ values of 30.4°, 35.7°, 43.4°, 53.9°, 57.4°, and 62.9° are in turn indexed as (2 2 0), (3 1 1), (4 0 0), (4 2 2), (5 1 1), and (4 4 0) crystal planes as the spinel type cubic structure of NiFe_2_O_4_ (ICSD card No. 10–0325) (Naidu and Narayana, 2019) (Figures 3A–C). As depicted in Figures 3B,C, the indicative diffraction peaks emerged at 2θ values of 31.6°, 34.3°, 36.3°, 47.4°, 56.5°, 62.9°, 66.4°, 67.8°, 69.1°, and 76.9° were matched up with (1 0 0), (0 0 2), (1 0 1), (1 0 2), (1 1 0), (1 0 3), (2 0 0), (1 1 2), (2 0 1), and (2 0 2) crystal planes of the hexagonal wurtzite structure of ZnO (ICSD card No. 36–1451) (Sharma et al., 2016). In the XRD pattern of ZnO/NiFe_2_O_4_/Co_3_O_4_ (Figure 3C), the existence of the characteristic peaks of ZnO and NiFe_2_O_4_ along with the appearance of diffraction peaks at 2θ° = 31.3°, 36.9°, 44.9°, 59.6°, and 65.3°, which was respectively assigned to (2 2 0), (3 1 1), (4 0 0), (5 1 1), and (4 4 0) reflection planes of the cubic spinel structure of Co_3_O_4_ (ICSD card No. 09–0418) (Wu et al., 2010), proved the successful preparation of the photocatalyst.

#### 3.1.3 X-ray photoelectron spectroscopy analysis

**FIGURE 4:**
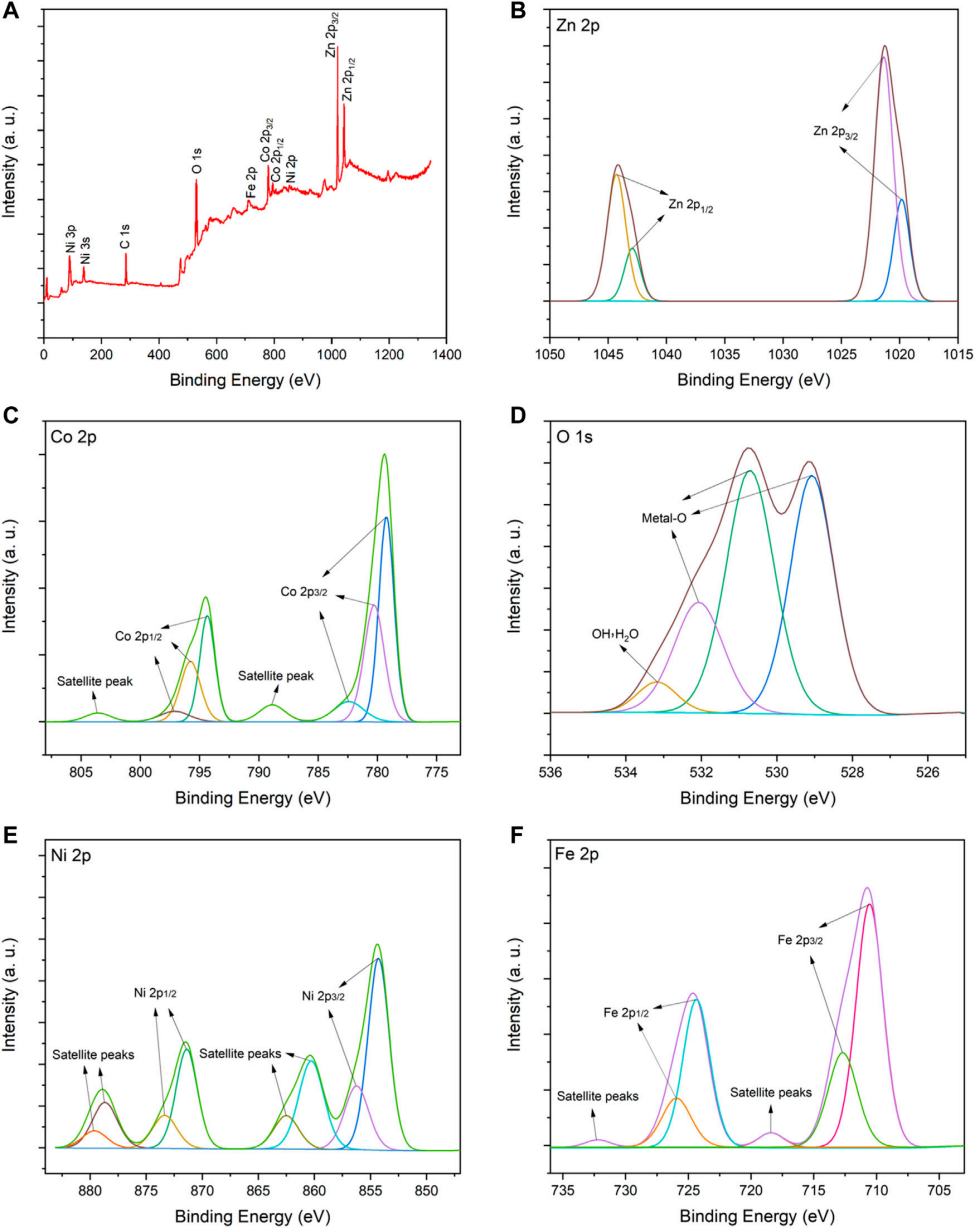
**(A)** XPS elemental survey spectrum, and high-resolution XPS spectra for **(B)** Zn 2p, **(C)** Co 2p, **(D)** O 1s, **(E)** Ni 2p and **(F)** Fe 2p of ZnO/NiFe_2_O_4_/Co_3_O_4_.

The element valence state for ZnO/NiFe_2_O_4_/Co_3_O_4_ is characterized *via* the XPS analysis (Figure 4). XPS elemental survey of ZnO/NiFe_2_O_4_/Co_3_O_4_ clearly confirms the presence of Zn, Co, O, Ni, and Fe elements in the photocatalyst structure (Figure 4A). As presented in Figure 4B, the high-resolution X-ray photoelectron spectroscopy (XPS) spectrum of Zn 2p is resolved into four peaks located at 1019.8 and 1021.3 eV for Zn 2p_3/2_, and 1042.9 and 1044.2 eV for Zn 2p_1/2_. These peaks are related to the zinc lattice in ZnO (Srivastava et al., 2018; Bharti et al., 2021; Kohantorabi et al., 2021). In addition, the observed spin-orbital split of Zn (between Zn 2p_1/2_ and Zn 2p_3/2_) is about 23 eV, which is in agreement with the reference value of ZnO (Liu et al., 2019; Kohantorabi et al., 2021). These results indicated the presence of Zn with +2 oxidation state. Figure 4C displays the high-resolution XPS spectrum of Co 2p with two sets of peaks corresponding to Co 2p_3/2_ and Co 2p_1/2_. The fitting peaks with the binding energies of 779.2, 794.3, and 797.1 eV are certified to Co^3+^, while the other three fitting peaks (780.2, 782.4, and 795.7 eV) belong to Co^2+^. Two satellite peaks at 788.8 and 803.5 eV are also consistent with Co 2p _3/2_ and Co 2p _1/2_, respectively (Tan et al., 1991; Duan and Chen, 2017; Guo et al., 2018). The high-resolution O 1s spectrum in Figure 4D can be further deconvoluted into four fitting peaks at 529.07, 530.70, 532.09 (attributed to crystal lattice oxygen molecules), and 533.17 eV (attributed to physically adsorbed H_2_O and oxygen on the surface of the catalyst) (Zhang et al., 2019; Kohantorabi et al., 2021; Ravichandran et al., 2021). Figure 4E shows the high-resolution XPS spectrum for Ni 2p. The main binding energy peaks of Ni 2p_3/2_ (at 854.2 and 856.2) and Ni 2p_1/2_ (at 871.4 and 873.3 eV) can demonstrate the presence of Ni^2+^ species. Also, the satellite peaks at 860.3 and 862.5 eV, which are consistent with Ni 2p_3/2_ and two others at 878.6 and 879.5 eV, which are compatible with Ni 2p_1/2_, could further confirm the existence of Ni with +2 oxidation state (Li et al., 2015; Yadav et al., 2019; Kang et al., 2020). In the high-resolution XPS spectrum of Fe 2p (Figure 4F), the peaks located at 710.5 and 712.7 eV are assigned to Fe 2p_3/2_, while the peaks centered at 724.3 and 725.9 are allocated to Fe 2p_1/2_. These observations, besides the satellite peaks at 718.4 (Fe 2p_3/2_) and 732.2 (Fe 2p_1/2_), revealed that the Fe cation has the oxidation state of +3 in the catalyst (Pham et al., 2014; Suresh et al., 2014).

#### 3.1.4 UV–vis diffuse reflectance spectroscopy analysis

**FIGURE 5:**
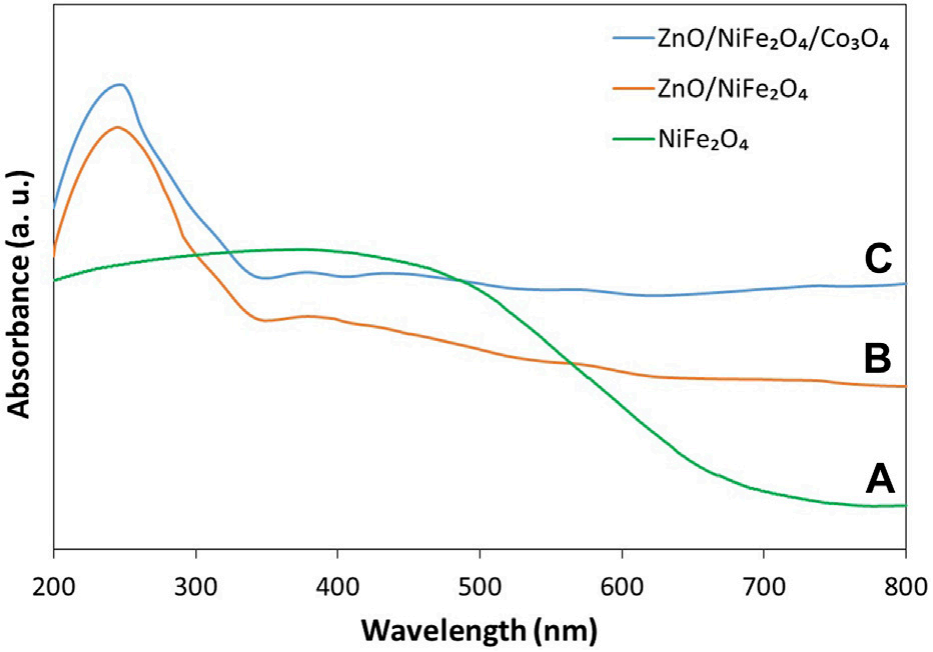
DRS analysis of **(A)** NiFe_2_O_4_, **(B)** ZnO/NiFe_2_O_4_ and **(C)** ZnO/NiFe_2_O_4_/Co_3_O_4_.

UV-vis diffuse reflectance spectroscopy (DRS) technique was conducted to measure the optical absorption properties of NiFe_2_O_4_, ZnO/NiFe_2_O_4_ and ZnO/NiFe_2_O_4_/Co_3_O_4_ (Figure 5). The results revealed that NiFe_2_O_4_ has a good ability to absorb light in the visible area (Figure 5A). As ZnO can predominantly absorb the UV light (below 400 nm), the combination of NiFe_2_O_4_ and ZnO can enhance the absorptive capacity in the visible light region, as well as the UV area (Figure 5B). Interestingly, the incorporation of Co_3_O_4_ to the ZnO/NiFe_2_O_4_ could effectually improve the optical capability of the photocatalyst. As exhibited in Figure 5C, the ZnO/NiFe_2_O_4_/Co_3_O_4_ photocatalyst has a boosted and strong light response in the entire UV and visible light region (200–800 nm).

**FIGURE 6:**
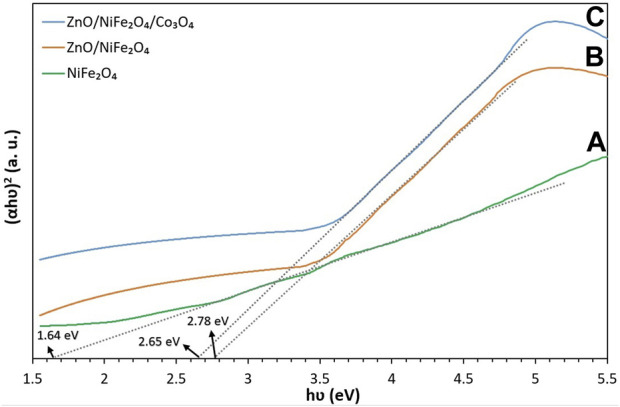
Tauc plot of **(A)** NiFe_2_O_4_, **(B)** ZnO/NiFe_2_O_4_ and **(C)** ZnO/NiFe_2_O_4_/Co_3_O_4_.

The band gap energies of NiFe_2_O_4_, ZnO/NiFe_2_O_4_ and ZnO/NiFe_2_O_4_/Co_3_O_4_ were calculated based on Tauc formula as given below:

$${\left(\alpha h\upsilon \right)}^{n}=K\left(h\upsilon -Eg\right)$$

where “hυ” is the photon energy, “α” is the absorption coefficient, “K” is a constant, and “n” is either 2 for direct transition or ½ for an indirect transition. The Tauc diagram of (αhυ)^2^ *vs*. hυ (Figure 6) revealed that the band gap energies of NiFe_2_O_4_, ZnO/NiFe_2_O_4_ and ZnO/NiFe_2_O_4_/Co_3_O_4_ are 1.64, 2.78, and 2.65 eV, respectively. The second power (*n* = 2) was used for this calculation as each of the constituent components of the ZnO/NiFe_2_O_4_/Co_3_O_4_ are well known to have a direct allowed transition (Viezbicke et al., 2015; Shi et al., 2019; Babu and Reddy, 2020).

These findings refer to the supreme capability of the ZnO/NiFe_2_O_4_/Co_3_O_4_ photocatalyst to separate the photo-induced electron and hole pairs efficiently and suggested that ZnO/NiFe_2_O_4_/Co_3_O_4_ could be used as a promising photocatalyst under solar light irradiation.

#### 3.1.5 Vibrating sample magnetometer analysis

The magnetic behavior of ZnO/NiFe_2_O_4_/Co_3_O_4_ was exhibited *via* the Vibrating sample magnetometer (VSM) analysis at room temperature (Supplementary Figure S1). The measured saturation magnetization value of the catalyst was found to be around 32.38 emu/g. No hysteresis loop is specified in the catalyst magnetization curve, which indicates its superparamagnetic properties.

#### 3.1.6 Morphology analysis

**FIGURE 7:**
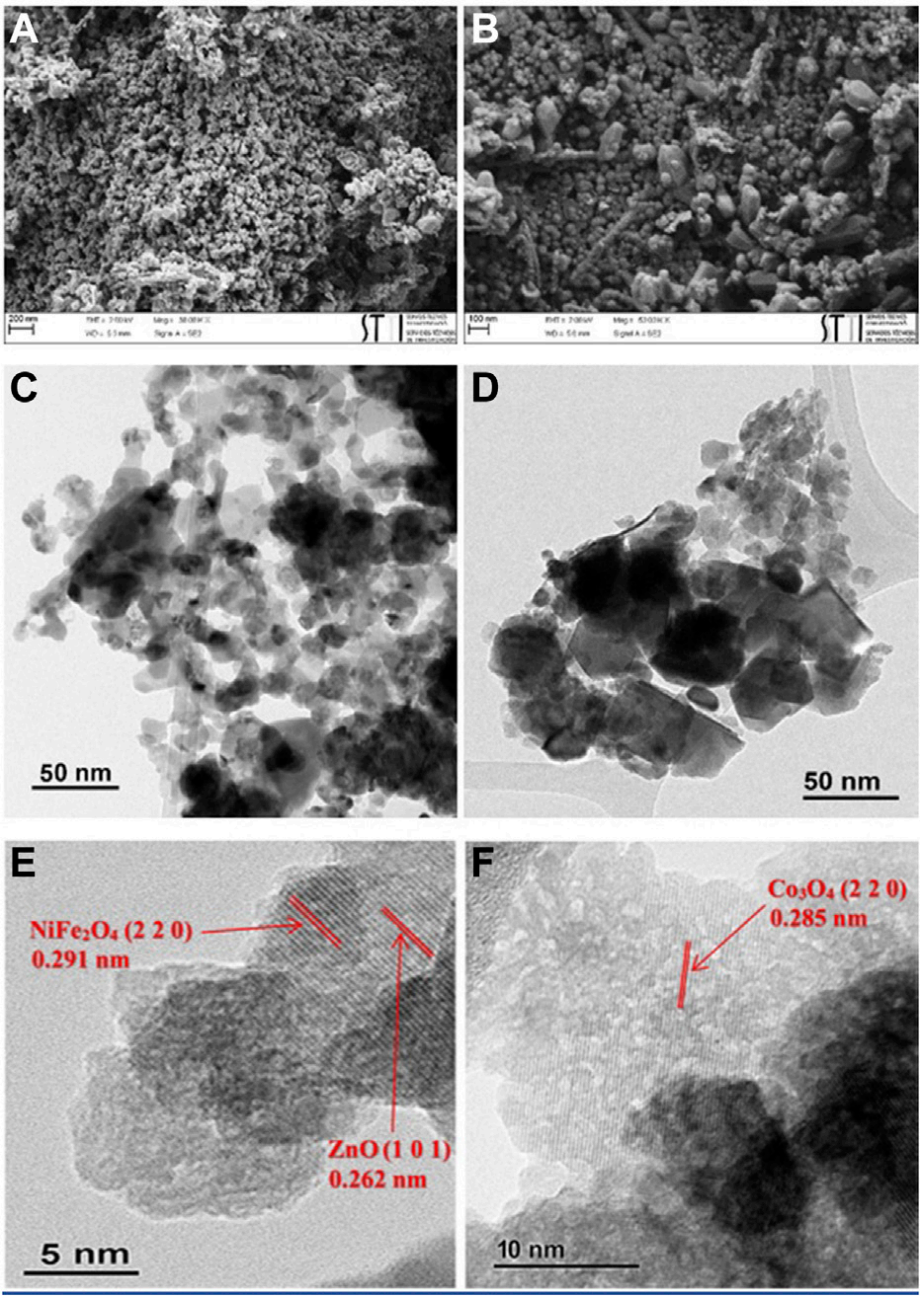
**(A,B)** FESEM of ZnO/NiFe_2_O_4_/Co_3_O_4_, **(C,D)** TEM of ZnO/NiFe_2_O_4_/Co_3_O_4_, and (**E,F**) HRTEM of ZnO/NiFe_2_O_4_/Co_3_O_4_.

To investigate the structural morphology of ZnO/NiFe_2_O_4_/Co_3_O_4_, FESEM and TEM analyses were performed, and the results are shown in Figure 7. It can be deduced from the FESEM images that ZnO has a hexagonal structure and NiFe_2_O_4_ and Co_3_O_4_ have cubic structures, with satisfying monodispersity (Figures 7A,B). TEM images (Figures 7C,D) corroborated the above-mentioned structures and disclosed that the mean sizes of ZnO particles were about 25–40 nm, while the particle sizes of NiFe_2_O_4_ and Co_3_O_4_ were measured to be between 10 and 25 nm. In addition, the HRTEM images show that the nanoparticles exhibit high crystallinity (Figures 7E,F), in which the lattice fringes of 0.262 nm (1 0 1), 0.291 nm (2 2 0), and 0.285 nm (2 2 0), could be attributed to ZnO, NiFe_2_O_4_, and Co_3_O_4_, respectively (Zhou et al., 2009; Zhang et al., 2012; Khosravi-Nejad, et al., 2019).

#### 3.1.7 Energy dispersive spectroscopy and elemental mapping analysis

The elemental composition and their thorough dispersion in the structure of ZnO/NiFe_2_O_4_/Co_3_O_4_ were further investigated *via* Energy dispersive spectroscopy (EDS) and elemental mapping analysis. As it is evident in (Supplementary Figure S2A), the characteristic peaks of Zn, O, Ni, Fe, and Co elements could be perceived from the EDS analysis of the catalyst. In the elemental mapping analysis of the catalyst (Supplementary Figures S2B−G), a uniform distribution of Zn, O, Ni, Fe, and Co atoms could be observed throughout the entire surface of the catalyst.

### 3.2 An investigation into the parameters affecting the photocatalytic degradation of TC in the presence of solar light

**FIGURE 8:**
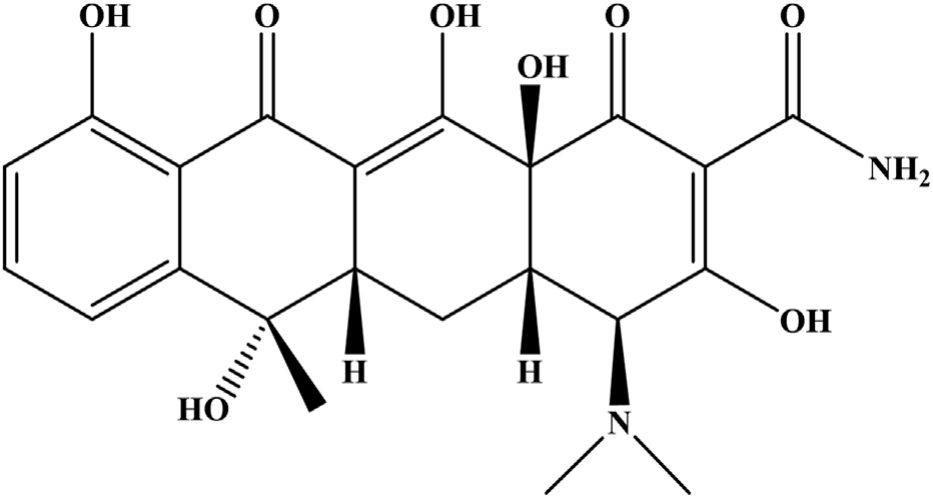
Molecular structure of TC.

Along with the industrial development of the world, the excessive release of harmful residues of antibiotics into the aquatic environment has seriously threatened the life of living ecosystems. TC (Figure 8), as one of the most widely used antibiotics in the world, plays an imperative role in the prevention and treatment of bacterial infections in humans and animals (Zhang et al., 2020). However, the elimination of TC *via* conventional wastewater treatments faces many challenges, which refers to the incomplete metabolization of TC in body and also in nature due to its low biodegradability (Boxall et al., 2003; Zhang et al., 2015). As far as we know, only a few reports are available which have taken the advantages of using direct sunlight as a natural source of energy for the degradation of TC through a photocatalytic pathway (Kumar et al., 2022; Liu et al., 2021; Panneri et al., 2017; Lallimathi et al., 2020; Dadigala et al., 2019; Sharma et al., 2020; Kumar et al., 2022; Ai et al., 2016; Jin et al., 2022). Most of these methods interfere with one or more of the following drawbacks, including the use of large quantities of the photocatalyst, low degradation efficiency, incomplete recovery of the photocatalyst, prolonged times, and the utilization of expensive/complex photocatalytic systems. Therefore, it is crucial to develop a more efficient, sustainable, and convenient method to deal with the problem of TC contamination.

**FIGURE 9:**
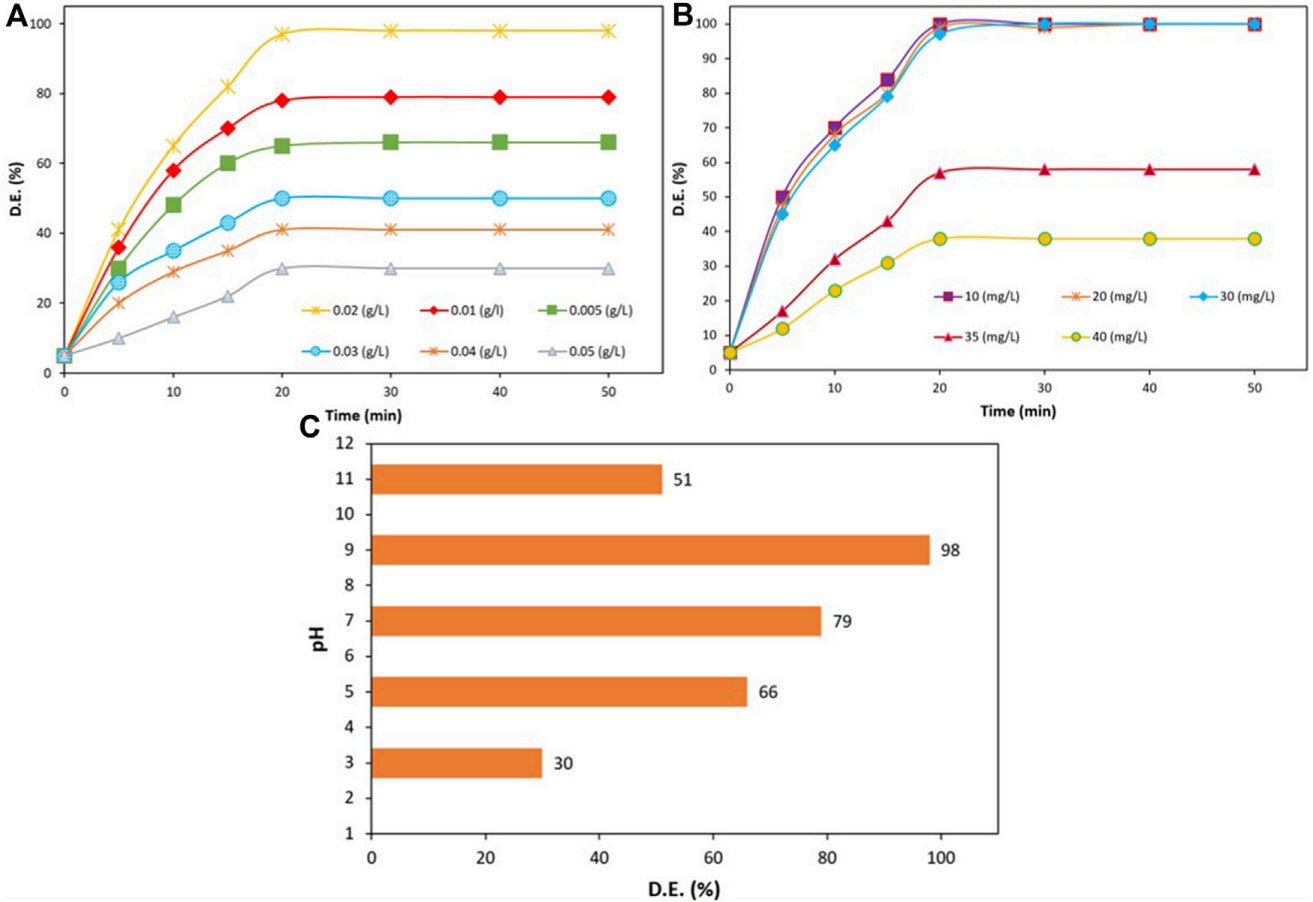
**(A)** The effect of photocatalyst amount on the efficiency of the TC photodegradation (TC amount = 30 mg L^−1^, and pH = 9), **(B)** TC concentration effect on the progress of the photodegradation process (photocatalyst amount = 0.02 g L^−1^, and pH = 9), **(C)** The effect of pH on TC degradation (TC concentration = 30 mg L^−1^, photocatalyst quantity = 0.02 g L^−1^, and irradiation time = 20 min).

The influence of various parameters, including the photocatalyst amount, primary concentration of TC, and pH quantity, on the photocatalytic degradation of TC was studied. Figure 9A depicted the effect of applying various amounts of ZnO/NiFe_2_O_4_/Co_3_O_4_ (0.005–0.05 g L^−1^) on TC photocatalytic degradation rate. As it can be perceived from the results, when the photocatalyst quantity was reached from 0.005 to 0.02 g L^−1^, the efficiency of the photodegradation process obviously improved from 65 to 98 within 20 min under sunlight illumination. This enhancement in the efficiency was mainly ascribed to the increment in the amount of the active catalytic sites, which consequently promotes the appropriate interactions between the photocatalyst and the TC. However, since the light scattering was disturbed due to the solution turbidity in higher concentrations of the photocatalyst (Sohrabi and Akhlaghian, 2016) (0.5 g L^−1^), a rate drop in the photodegradation was observed. Therefore, 0.02 g L^−1^ was opted as the best concentration of the catalyst for the photocatalytic degradation of TC (30 mg L^−1^).

In the following, various concentrations of TC (10, 20, 30, 35, and 40 mg L^−1^) were examined for monitoring the effect of the initial amount of TC on the photocatalytic degradation progress over ZnO/NiFe_2_O_4_/Co_3_O_4_. It is evident in Figure 9B that an excellent degradation efficiency was resulted in lower TC concentrations (10–30 mg L^−1^). On the other hand, by further increasing the TC initial concentration (above 30 mg L^−1^), the rate of reaction intensively declined. This observation implies the fact that the rate of photocatalytic reactions initially rises with an increase in catalyst loading and then declines at a certain value owing to the decrease in the light scattering and non-uniform light intensity distribution. Hence, at higher concentrations, the extra amounts of the TC molecules were accumulated on the photocatalyst surface, which disturbs the appropriate photocatalytic performance (Arghavan et al., 2021) and leads to unsatisfying degradation results. According to these findings, the optimal TC degradation amount was achieved to be 30 mg L^−1^.

The contact time effect was also investigated. Figure 9B demonstrated that the progress of the degradation process noticeably increased up to 20 min. However, over more time, the TC degradation process no longer progressed, which could be due to the reason that not adequate active radicals remain in the solution. Based on the results obtained, 0.02 g/L was chosen as the best catalyst concentration for the photocatalytic degradation of 30 mg/L TC (in 20 min).

In the next experiment, the effect of pH variation (3–11) on the photocatalytic degradation of TC (30 mg L^−1^) in the presence of ZnO/NiFe_2_O_4_/Co_3_O_4_ (0.020 g L^−1^) was investigated under the solar light irradiation (Figure 9C). As it is evident from the plot of pH variation vs. the radiation time, the TC degradation efficiency was considerably increased by the pH enhancement and reached its maximum (98%) at a pH of 9. However, the dramatically dropped degradation rate in acidic and further alkaline media can be due to the inappropriate interactions of antibiotic and ZnO/NiFe_2_O_4_/Co_3_O_4_ in such solutions (Isari et al., 2020; Nasseh et al., 2020; Xu et al., 2020).

**FIGURE 10:**
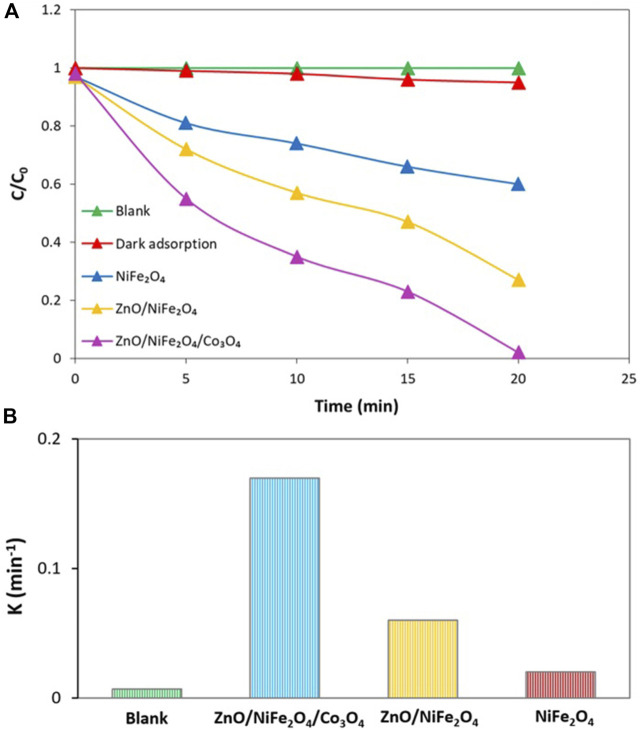
**(A)** Sunlight driven photocatalytic degradation of TC under optimal conditions, **(B)** The degradation rate constants for the sunlight driven photocatalytic degradation of TC under optimal conditions. (The “k” values are obtained according to the kinetics done in the next section).

To have a deep insight into the efficient photocatalytic performance of ZnO/NiFe_2_O_4_/Co_3_O_4_ in the sunlight driven photocatalytic degradation of TC, control tests were performed in optimized conditions (Figure 10A). The blank test exhibited that there is no progress in the TC photodegradation process without using any photocatalyst. Furthermore, the evaluation of the possible capability of ZnO/NiFe_2_O_4_/Co_3_O_4_ for TC adsorption under dark conditions is less than 5%. Thereafter, the activity of NiFe_2_O_4_ and ZnO/NiFe_2_O_4_ were tested simultaneously towards the photocatalytic degradation of TC under the same conditions, and the results indicated that the performance of ZnO/NiFe_2_O_4_/Co_3_O_4_ was much higher compared to that of NiFe_2_O_4_ and ZnO/NiFe_2_O_4_. After 20 min of the degradation reaction under optimal conditions, about 98% of TC was degraded by ZnO/NiFe_2_O_4_/Co_3_O_4_, while the degradation efficiencies were calculated to be 40% and 73% for NiFe_2_O_4_ and ZnO/NiFe_2_O_4_, respectively (Figure 10A). The obtained reaction rate for TC photocatalytic degradation over ZnO/NiFe_2_O_4_/Co_3_O_4_ was about 8.5 and 2.8 times higher than that of NiFe_2_O_4_ and ZnO/NiFe_2_O_4_, respectively (Figure 10B). It is assumed that the synergistic optical effects between ZnO, NiFe_2_O_4_, and Co_3_O_4_ resulted in the significant separation of charge carriers and in parallel reduced the speed of the recombination of photogenerated electrons and holes. This phenomenon can be responsible for the enhanced photocatalytic degradation ability of the photocatalyst.

### 3.3 Total organic carbon analysis

**FIGURE 11:**
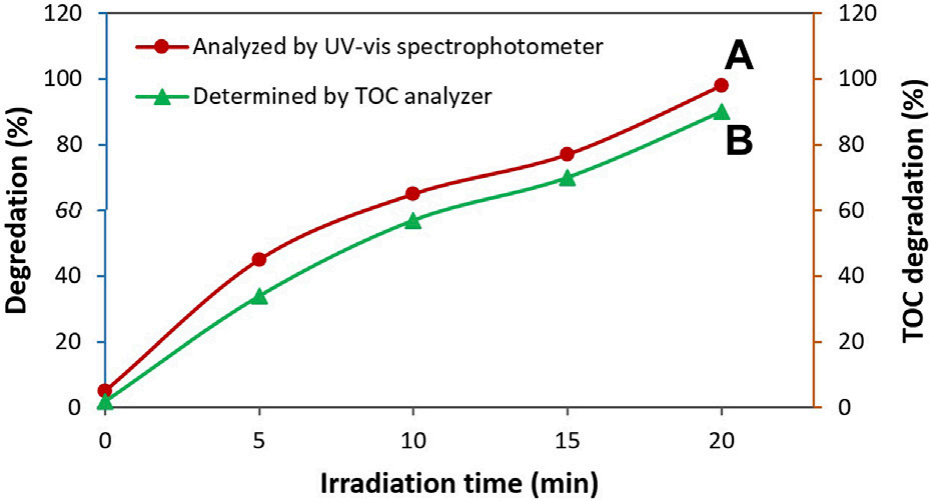
Degradation rate of TC using ZnO/NiFe_2_O_4_/Co_3_O_4_ obtained from the absorbance measurements **(A)** and TOC analysis **(B)** versus the irradiation time.

For quantitatively monitor the mineralization process of TC under the optimized conditions, total organic carbon (TOC) analysis was performed. The percentage of TOC degradation can be determined by using the following equation:

$$TOC\;degradation\;\left(\%\right)=\frac{{\left(TOC\right)}_{0}-{\left(TOC\right)}_{t}}{{\left(TOC\right)}_{0}}\times 100$$

where (TOC)_0_ is related to the initial TOC of the TC solution and (TOC)_t_ is devoted to the TOC of the TC solution at specific reaction time during treatment with photocatalyst. As it is illustrated in Figure 11, the capability of the TC degradation measured from the TOC analysis revealed a trend similar to the data obtained by the UV-vis study. In fact, almost complete mineralization of TC antibiotic was achieved (percentage of TOC degradation = 90%). These results more endorsed the efficiency of the presented photocatalyst for TC degradation.

### 3.4 Kinetic study

Based on the Langmuir–Hinshelwood (L-H) kinetic model, an effectual photocatalytic degradation approach well follows the pseudo-first-order kinetics (Ahmad and Mondal, 2012; Alvarez-Ramirez et al., 2016) with respect to the below equation:

ln $\left(\frac{C_{0}}{C_{t}}\right)=-kt$

where C_0_ and C_t_ are in turn the initial concentration (mg L^−1^) of the contaminant at the inception of the process, and the remaining concentration (mg L^−1^) of the contaminant at time t. t shows the particular reaction time (min), and k is the rate constant.

**FIGURE 12:**
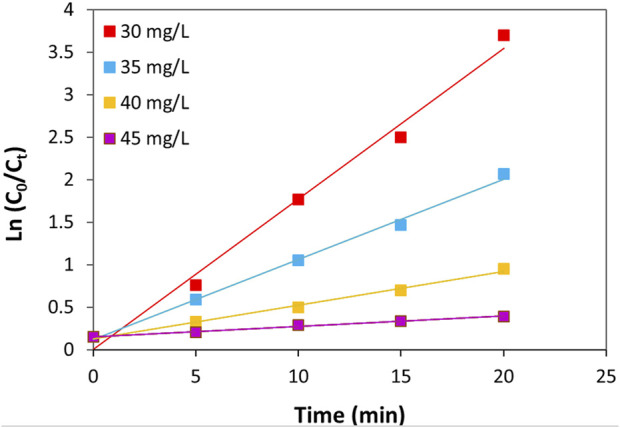
Plot of ln (C_0_/C_t_) *vs*. time for different concentrations of TC (pH = 9 and catalyst amount = 0.02 g L^−1^).

The kinetics of TC photocatalytic degradation over ZnO/NiFe_2_O_4_/Co_3_O_4_ were evaluated by conducting some typical experiments with different concentrations of TC in optimized reaction conditions (catalyst amount = 0.02 g L^−1^, and pH = 9). Figure 12 shows a linear relationship between ln (C_0_/C_t_) and the irradiation times. Accordingly, it can be concluded that the photocatalytic degradation process of TC over the ZnO/NiFe_2_O_4_/Co_3_O_4_ evidently follows the pseudo-first-order kinetic model. Table 1 illustrates the kinetic details for the photocatalytic degradation of different concentrations of TC. As it is obvious in all experimented concentrations of TC, obtaining the coefficient of determination *R* ^2^) values close to 1 is a further evidence to approve the appropriateness of this process. It is interesting to note that by the increase of the TC concentration from 30 to 45 mg L^−1^, a decrease in the “k” values from 0.1768 to 0.0122 min^−1^ was observed. This could be related to the assumption that at higher concentrations of TC, the production of intermediates might be increased, which leads to reduce the degradation “k” values owing to the drop in the number of potent radicals in the solution.

**TABLE 1:** The kinetic parameters for the pseudo-first-order model of TC photocatalytic degradation at different concentrations.

Entry	TC initial concentration (mg L^−1^)	Equation	*R* ^2^	K (min^−1^)
1	30	y = 0.1768x + 0.008	0.9889	0.1768
2	35	y = 0.0944x + 0.122	0.9959	0.0944
3	40	y = 0.0394x + 0.132	0.9938	0.0394
4	45	y = 0.0122x + 0.154	0.9917	0.0122

### 3.5 Recycling study of ZnO/NiFe_2_O_4_/Co_3_O_4_ in the TC photocatalytic degradation process

From the view of the practical applications, the stability and recyclability of the photocatalyst are very important subjects. In this line, the recyclability of ZnO/NiFe_2_O_4_/Co_3_O_4_ was probed by the successive photodegradation experiments of TC under the optimal conditions. At the end of each cycle, the photocatalyst was isolated from the reaction solution with the aid of an external magnetic field, washed several times with water, and dried at 80 ^°^C for 2 h. As plotted in Supplementary Figure S3, it was found that even after eight runs of reuses, no apparent decrease was observed in the catalytic potential of ZnO/NiFe_2_O_4_/Co_3_O_4_.

**FIGURE 13:**
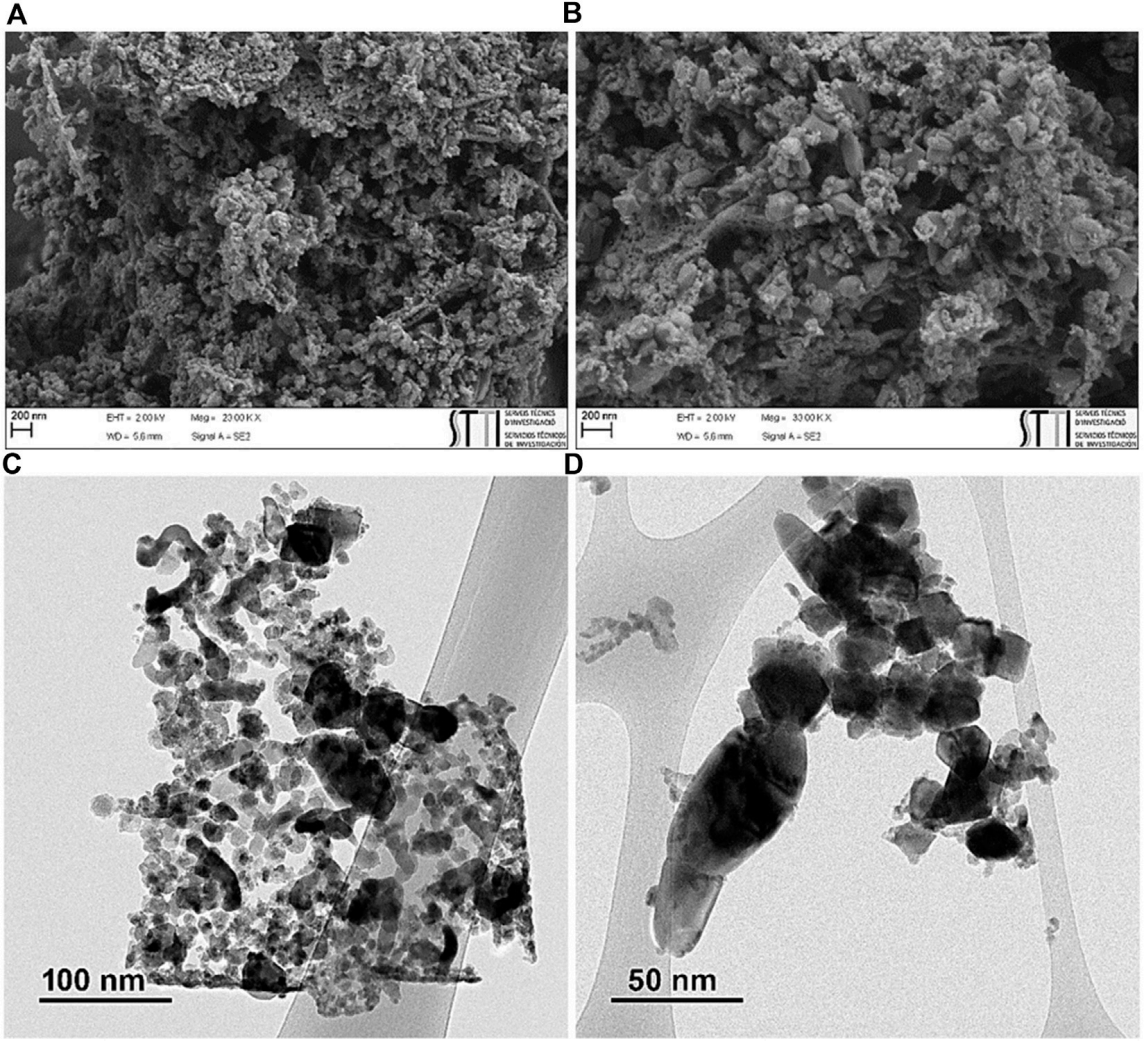
**(A,B)** FESEM of ZnO/NiFe_2_O_4_/Co_3_O_4_ and **(C,D)** TEM of ZnO/NiFe_2_O_4_/Co_3_O_4_ after eight times recycling.

Based on the FT-IR (Supplementary Figure S4), FESEM, and TEM (Figure 13) analysis, the chemical and morphological structure of ZnO/NiFe_2_O_4_/Co_3_O_4_ were almost entirely preserved after eight consecutive reuses, certifying the excellent stability of this photocatalyst for sunlight driven photocatalytic degradation of TC.

### 3.6 Comparative study

To further ensure the efficiency of the photocatalyst studied in this method, the advantages of this photocatalytic system were compared with all previously studied photocatalysts under natural sunlight and some recently reported procedures under visible light for TC degradation. (Table 2). The related findings clearly show the excellent photocatalytic potential of ZnO/NiFe_2_O_4_/Co_3_O_4_ and the high efficiency of the represented photocatalytic methodology for TC degradation. The superlative photocatalytic activity of ZnO/NiFe_2_O_4_/Co_3_O_4_ could correspond to the synergistic optical effects of ZnO, NiFe_2_O_4_, and Co_3_O_4_, which led to the significant separation of charge carriers and successfully sluggish the rate of the recombination of photo-induced electrons and holes.

**TABLE 2:** Comparison of the efficiency of ZnO/NiFe_2_O_4_/Co_3_O_4_ *vs*. all previously studied photocatalysts under natural sunlight and some recently reported procedures under visible light for TC degradation.

Entry	Photocatalyst	Catalyst dosage (g L^−1^)	TC concentration (mg L^−1^)	Light source	pH	Time (min)	Degradation efficiency (%)	References
1	ZnWO_4_/SnS_2_	0.6	20	Sunlight	—^a^	90	95	Kumar et al. (2022a)
2	MoO_3_/g-C_3_N_4_	0.05	10	Sunlight	—	100	85.2	Liu et al. (2021)
3	Urea-derived C_3_N_4_	0.5	44.4	Sunlight	—	120	93	Panneri et al. (2017)
4	CD^b^/CoFe_2_O_4_/g-C_3_N_4_	0.025	20	Sunlight	—	60	95	Lallimathi et al. (2020)
5	FeWO_4_/g-C_3_N_4_	0.05	20	Sunlight	—	90	88	Dadigala et al. (2019)
6	LaTiO_2_N/Bi_2_S_3_@RGO^c^	0.03	10	Sunlight	5	90	80.2	Sharma et al. (2020)
7	ZnWO_4_/Bi_2_MoO_6_/20 μl H_2_O_2_	0.6	20	Sunlight	—	90	95	Kumar et al. (2022b)
8	In_2_S_3_	2.5	20	Sunlight	—	30	99–100	Ai et al. (2016)
9	Ag_3_PO_4_/MIL100(Fe)	0.025	10	Xe lamp (350 W)	9	120	91.4	Xu et al. (2020)
10	Ni_2_FeO_4_/g-C_3_N_4_/1 ml H_2_O_2_	10	20	Visible LED lamp (40 W)	—	120	93	Palanivel and Mani, (2020)
11	Au modified MnFe_2_O_4_/50 mM H_2_O_2_	1	10	Xe lamp (300 W)	6	90	88.3	Qin et al. (2021)
12	LaNiO_3_/g-C_3_N_4_	0.1	20	Visible LED light (50 W)	7	120	88.1	Ghorai et al. (2022)
13	C–N–S tridoped TiO_2_	0.5	5	Xe arc lamp (150 W)	9	180	98	Wang et al. (2011)
14	Bi_2_Sn_2_O_7_-C_3_N_4_/Y^d^	1	20	Halogen lamp (400 W)	6	90	80.4	Heidari et al. (2018)
15	graphene-bridged Ag_3_PO_4_/Ag/BiVO_4_	0.5	10	Xe lamp (300 W)	5	60	94.96	Chen et al. (2017)
16	BiPO_4_/rGO/pg-C_3_N_4_	1	20	Xe lamp (300 W)	3	50	80	Xia et al. (2020)
17	Bi_m_O_n_Br_z_	0.2	25	Halogen lamp (400 W)	6	120	98.9	Khodaeipour et al. (2020)
18	ZnO/CeO_2_@HNTs^e^	0.3	20	Xe lamp (300 W)	8	60	87.25	Ye et al. (2016)
19	B-TiO_2_	0.2	10	Xe lamp (1000 W)	7	240	66.2	Wu et al. (2021)
20	ZnO/NiFe_2_O_4_/Co_3_O_4_	0.02	30	Sunlight	9	20	98	This work

^a^ This information is not mentioned in the article.

^b^ CD, carbon dot.

^c^ RGO, reduced graphene oxide.

^d^ Y, refers to the kind of zeolite.

^e^ HNTs, halloysite nanotubes.

### 3.7 Trapping of active species

The free radical/hole trapping experiments were conducted to find the main reactive species in the TC photocatalytic degradation (Supplementary Figure S5). Under the optimum conditions, the photocatalytic degradation of TC was conducted in the presence of isopropanol (IPA), benzoquinone (BQ), and ammonium oxalate (AO), which act in turn as the hydroxyl radical (^•^OH), superoxide radical (^•^O_2_^−^) and hole (h^+^) scavengers. As shown in Supplementary Figure S5, significant inhibition of the photocatalytic effect was observed after adding 1 mM of BQ or AO to the reaction mixture. This suggests that the photogenerated ^•^O_2_^−^ and h^+^ are the major reactive species responsible for this photodegradation process. A very small decrease in the progress of the process while using IPA (1 mM) shows that the contribution of ^•^OH in the photocatalytic process is weak.

## 4 Conclusion

In this study, ZnO/NiFe_2_O_4_/Co_3_O_4_ was fabricated and fully characterized by a series of methods. The molar ratio of ZnO/NiFe_2_O_4_/Co_3_O_4_ composite was obtained to be 1:0.75:0.5 based on the ICP analysis. FESEM and TEM analyses of the photocatalyt clearly affirmed the formation of a hexagonal structure of ZnO (25–40 nm) and the cubic structure of NiFe_2_O_4_ and Co_3_O_4_ (10–25 nm). Furthermore, the HRTEM images of the photocatalyst verified some key lattice fringes related to the photocatalyt structure. These data were in very good agreement with XRD analysis results. UV-vis DRS analysis and the corresponding Tauc plot of (αhν)^2^ *vs*. hν indicated that the photocatalyst has a narrow band gap energy (2.65 eV) with strong light response under solar light irradiation. The activity of the as-prepared photocatalyst was satisfactorily assessed for the photocatalytic degradation of TC under natural sunlight irradiation. The optimal degradation amount was acquired to be 98% for TC (30 mg L^−1^) in the presence of ZnO/NiFe_2_O_4_/Co_3_O_4_ (0.02 g L^−1^) within 20 min of reaction time in pH of 9, under the solar light illumination. It is noteworthy that the superb activity of the ZnO/NiFe_2_O_4_/Co_3_O_4_ might be assigned to the synergistic optical effects of ZnO, NiFe_2_O_4_, and Co_3_O_4_, resulting in the substantial separation of charge carriers, which has a direct influence on reducing the recombination speed of the photogenerated electrons/holes species. Based on the radical quenching experiment, the major involved reactive species in this process were evaluated to be the ^•^O_2_^−^ and h^+^. Moreover, it was observed that the photocatalytic degradation reaction probed in this investigation follows the pseudo-first-order kinetics, which points to the high efficiency of the photocatalyst. This study proposed a highly efficient, facile, and low-cost photocatalytic method to degrade TC by using a very low amount of the photocatalyst under natural sunlight source, within a short reaction time, in an air atmosphere. One of the most important aspects of this protocol is the convenient magnetic isolation (saturation magnetization of the photocatalyst was 32.38 emu/g based on VSM analysis) and reuse of the photocatalyst up to at least eight successive runs, which further highlights the operational applicability of this approach. Remarkably, the presented method has the preferred performance among the very few approaches reported for the photocatalytic degradation of TC under natural sunlight. This study can provide inspiration for sustainable environmental remediation of a broad range of contaminants.

## Data Availability

The original contributions presented in the study are included in the article/Supplementary Materials, further inquiries can be directed to the corresponding author.
