# Corrigendum: Solar light induced photocatalytic degradation of tetracycline in the presence of ZnO/NiFe_2_O_4_/Co_3_O_4_ as a new and highly efficient magnetically separable photocatalyst

**DOI:** 10.3389/fchem.2022.1090715

**Published:** 2022-11-18

**Authors:** Mohammadreza Doosti, Roya Jahanshahi, Shaghayegh Laleh, Sara Sobhani, José Miguel Sansano

**Affiliations:** ^1^ Department of Civil Engineering, Faculty of Engineering, University of Birjand, Birjand, Iran; ^2^ Department of Chemistry, College of Sciences, University of Birjand, Birjand, Iran; ^3^ Departamento de Química Orgánica, Facultad de Ciencias, Centro de Innovación en Química Avanzada (ORFEO-CINQA) and Instituto de Síntesis Orgánica (ISO), Universidad de Alicante, Alicante, Spain

**Keywords:** wastewater treatment, solar light, photocatalysis, heterogeneous catalysis, magnetically separable, tetracycline, degradation

In the original article, there was an error in the **Correspondence**, page 1. The author’s academic email address should have been included. The correct email addresses appear above.

The authors apologize for this error and state that this does not change the scientific conclusions of the article in any way. The original article has been updated.

