# Supplementary figures and images for: Solar light induced photocatalytic degradation of tetracycline in the presence of ZnO/NiFe2O4/Co3O4 as a new and highly efficient magnetically separable photocatalyst
Source: Front Chem. 2022 Oct 13;10:1013349. doi: 10.3389/fchem.2022.1013349 (PMC9606596; doi:10.3389/fchem.2022.1013349)

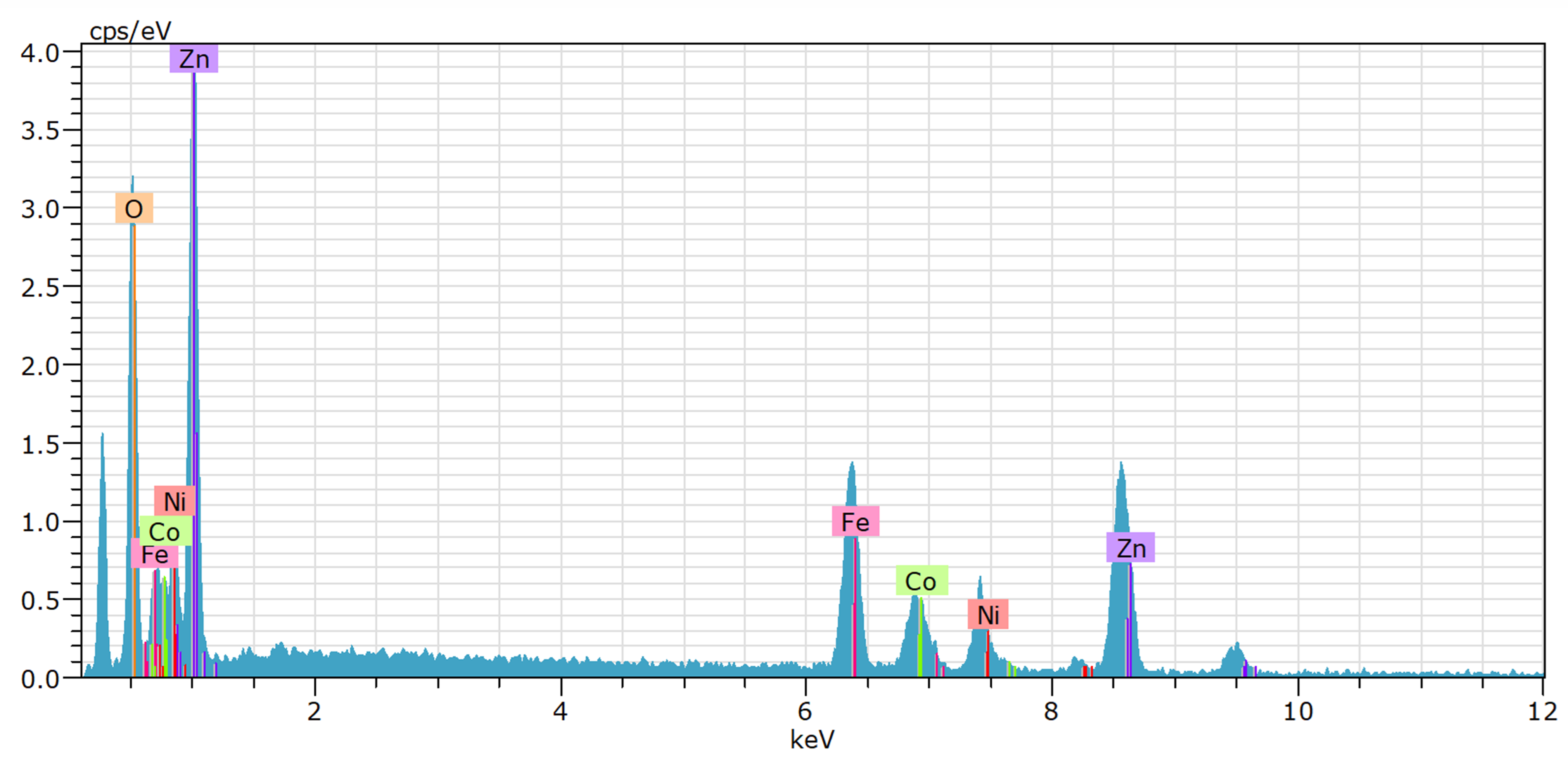

Supplement: Supplementary file 1 [file DataSheet2.zip › S2/S2, A.tif]

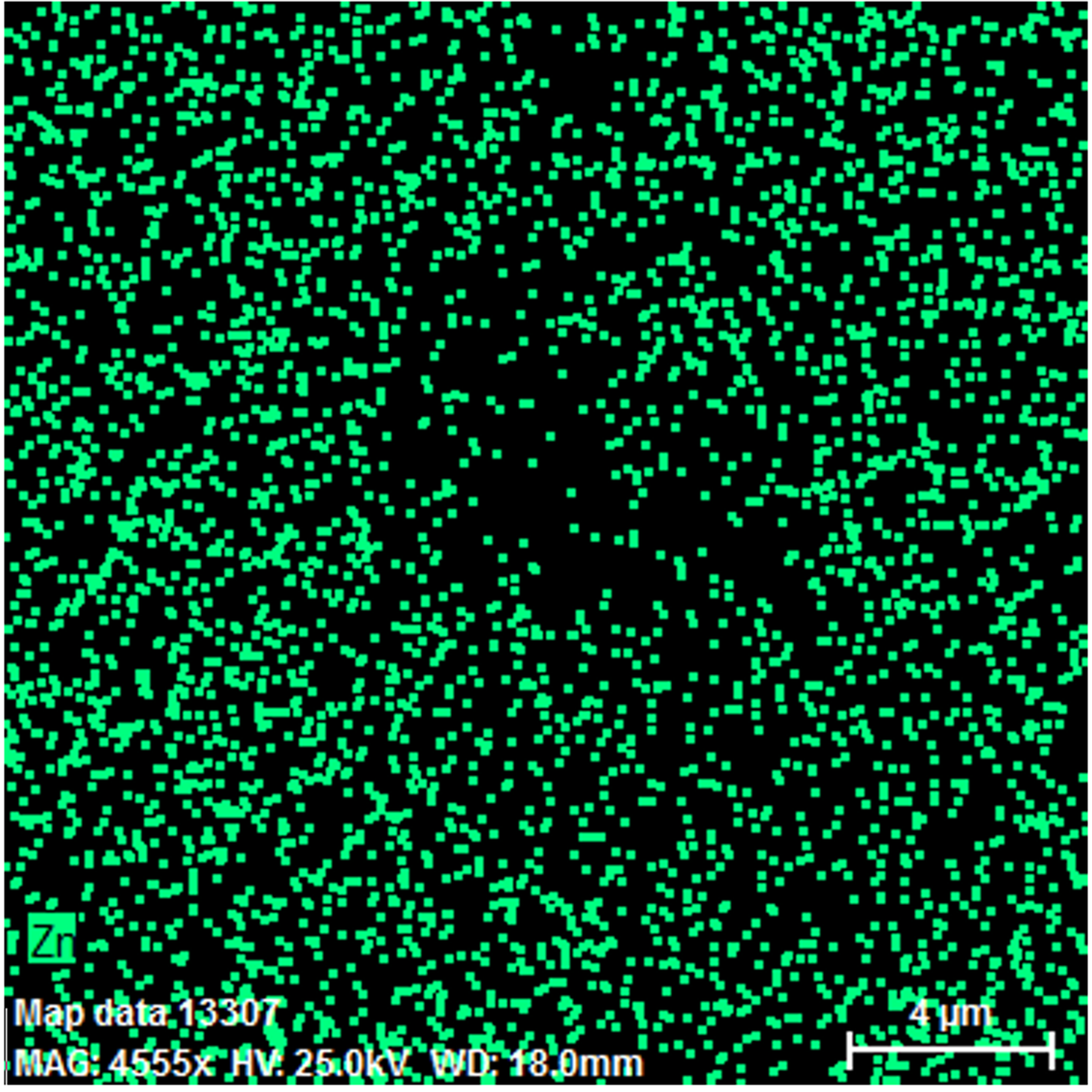

Supplement: Supplementary file 1 [file DataSheet2.zip › S2/S2, B.tif]

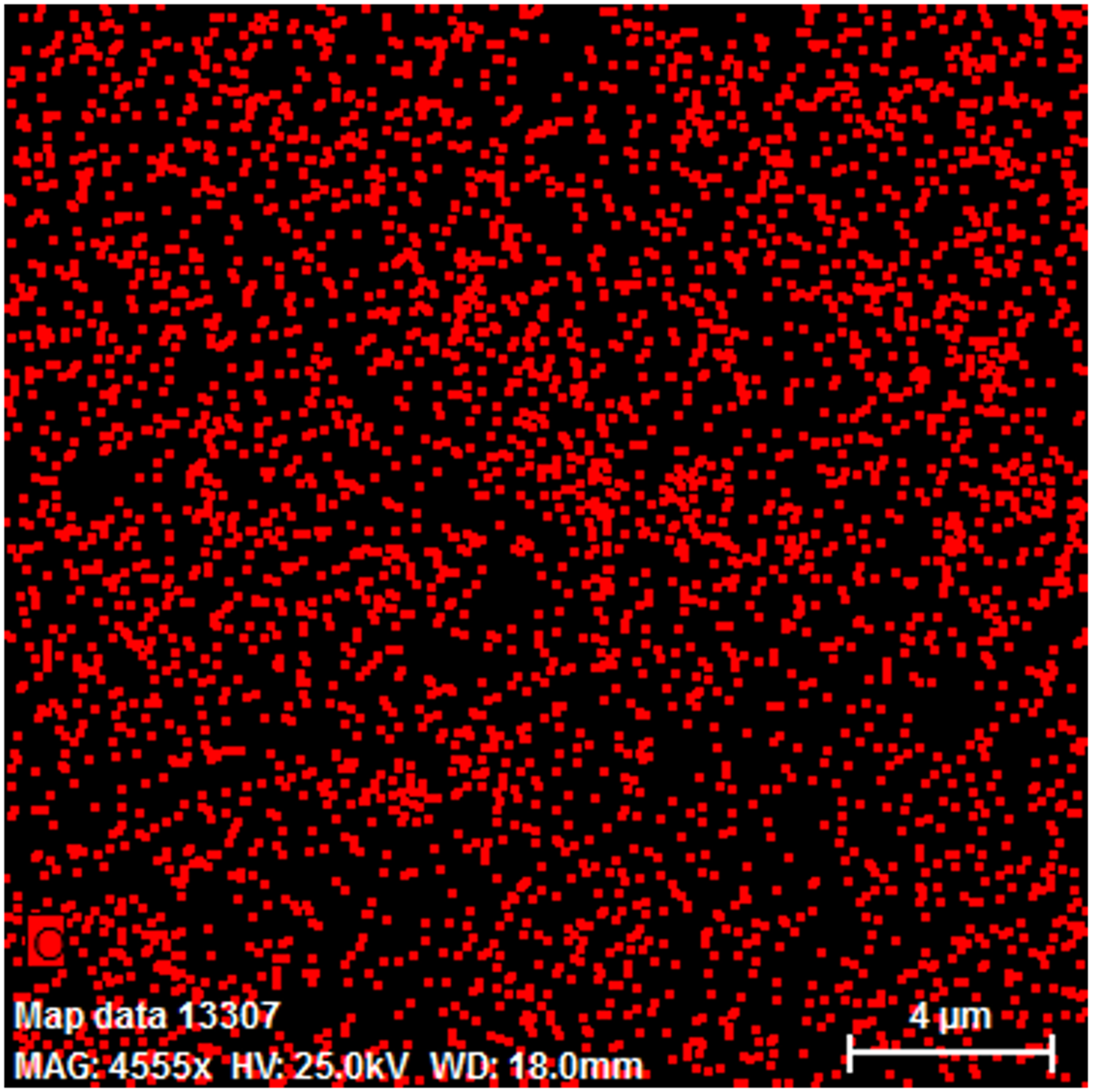

Supplement: Supplementary file 1 [file DataSheet2.zip › S2/S2,C.tif]

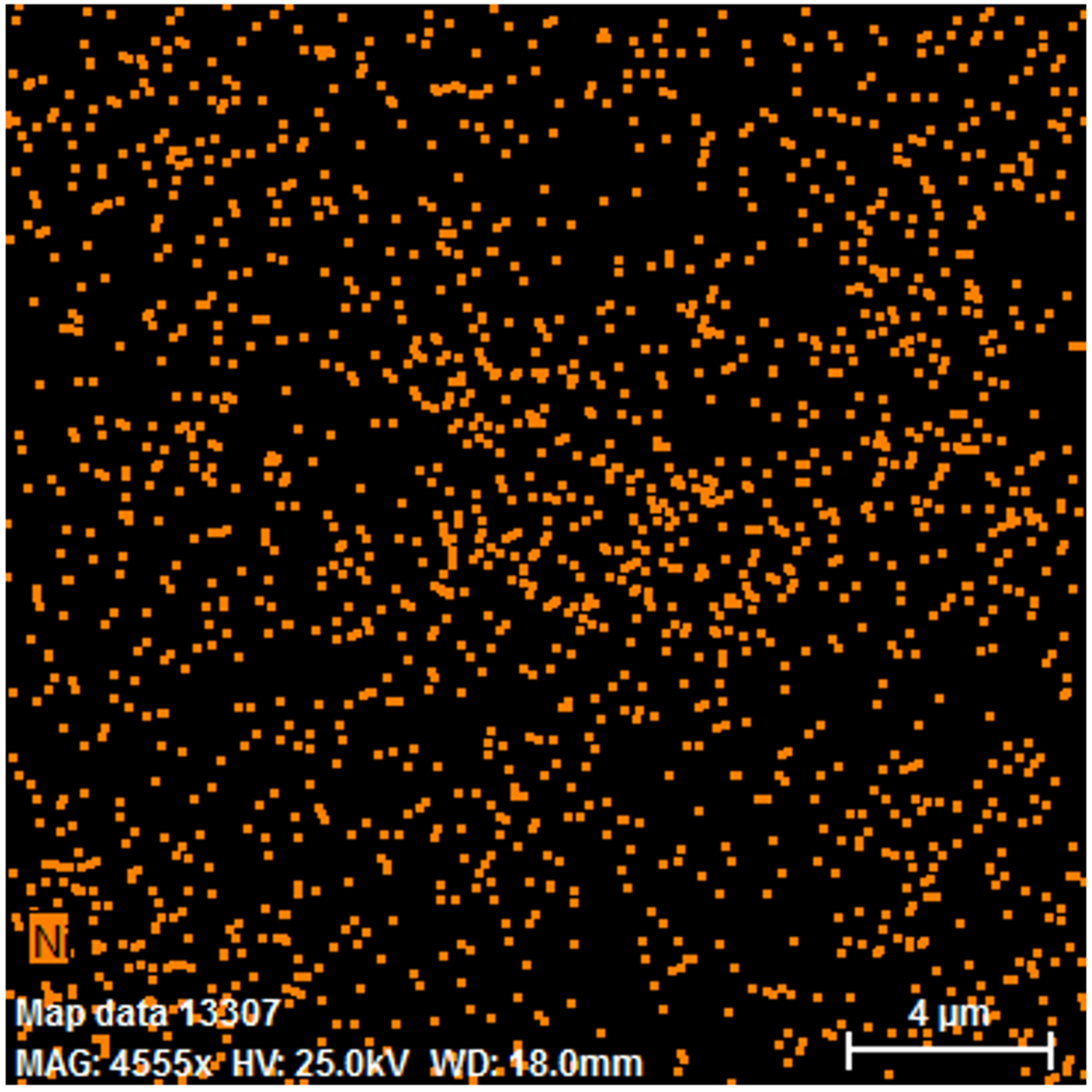

Supplement: Supplementary file 1 [file DataSheet2.zip › S2/S2,D.tif]

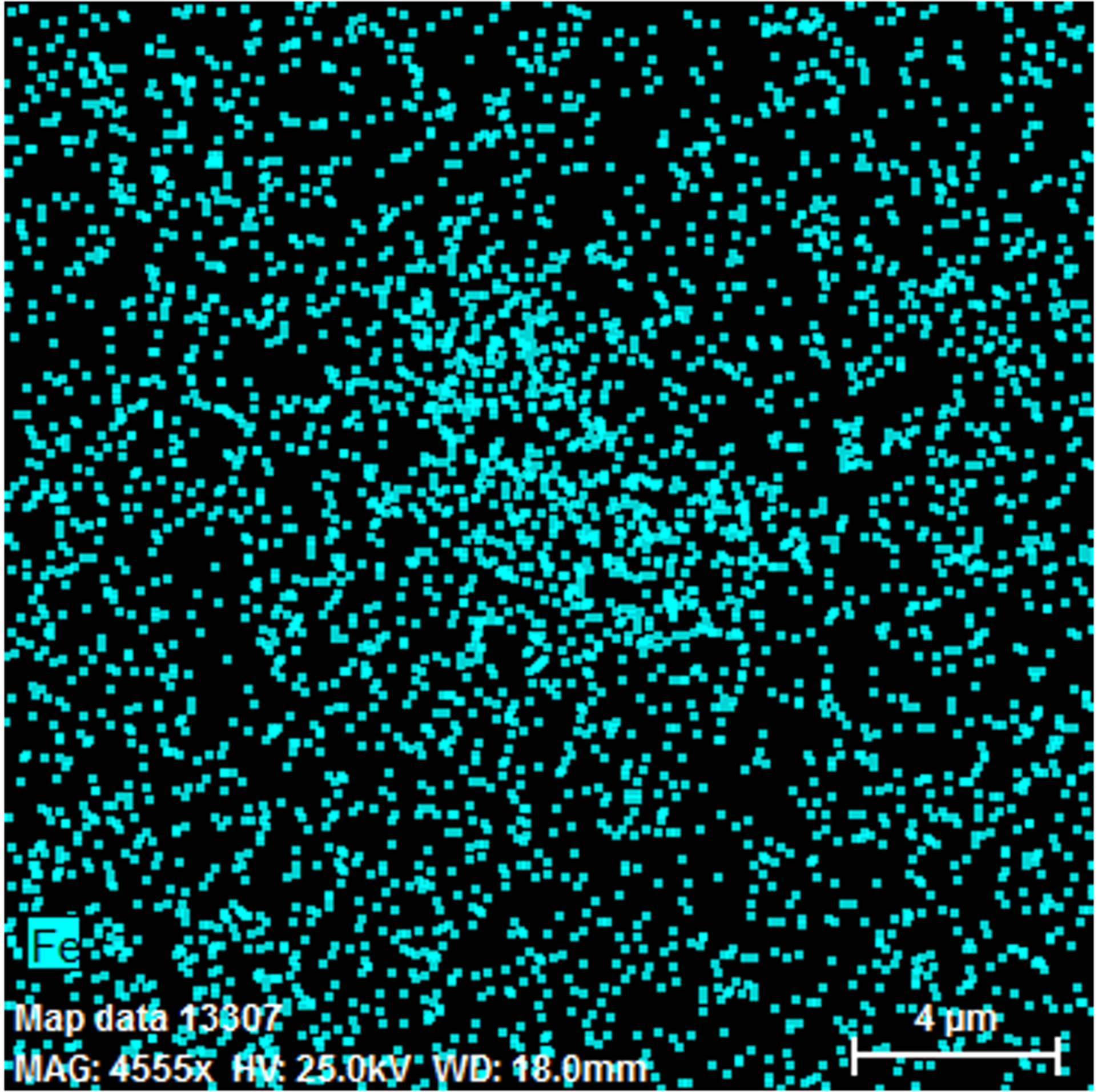

Supplement: Supplementary file 1 [file DataSheet2.zip › S2/S2,E.tif]

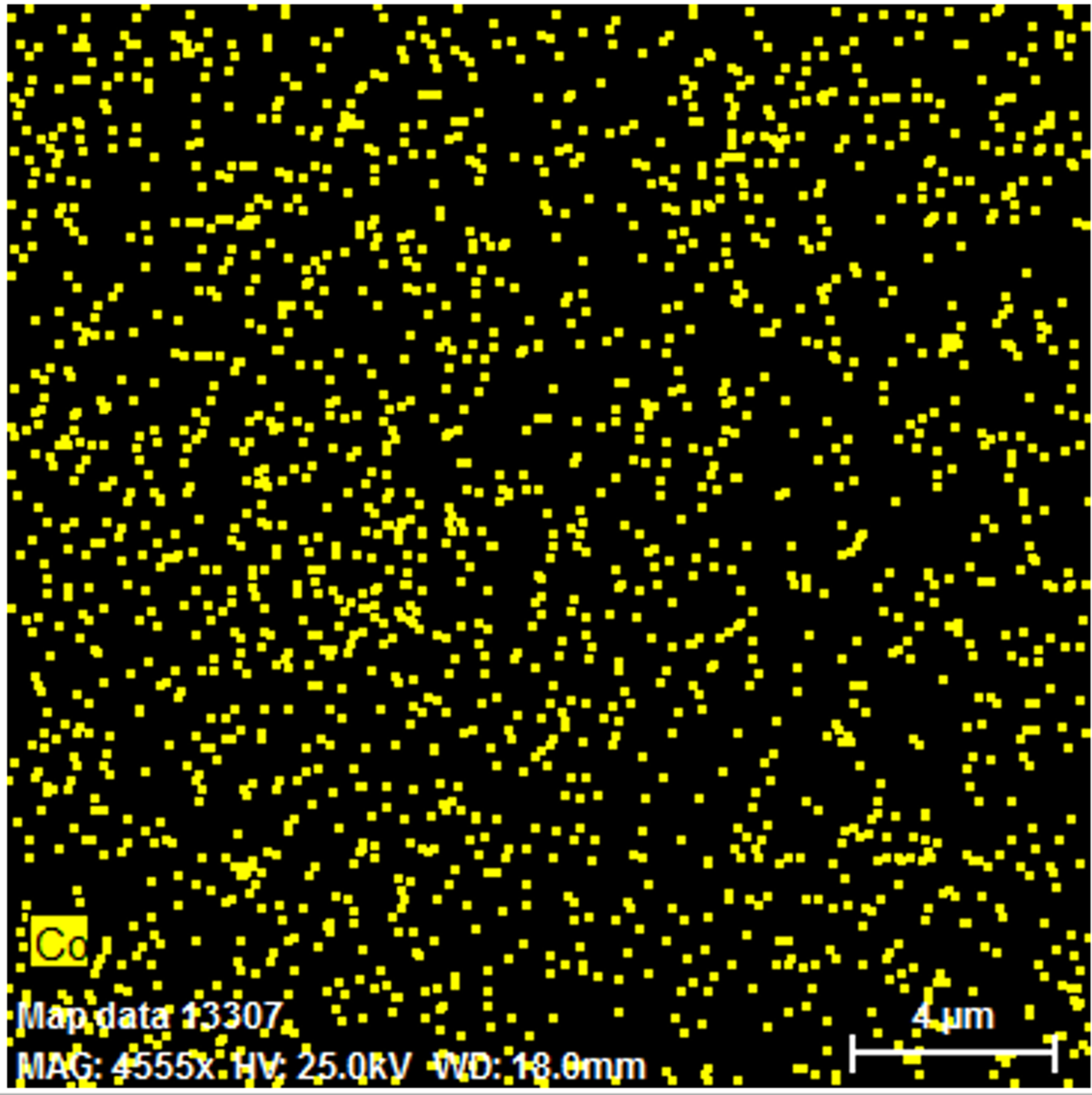

Supplement: Supplementary file 1 [file DataSheet2.zip › S2/S2,F.tif]

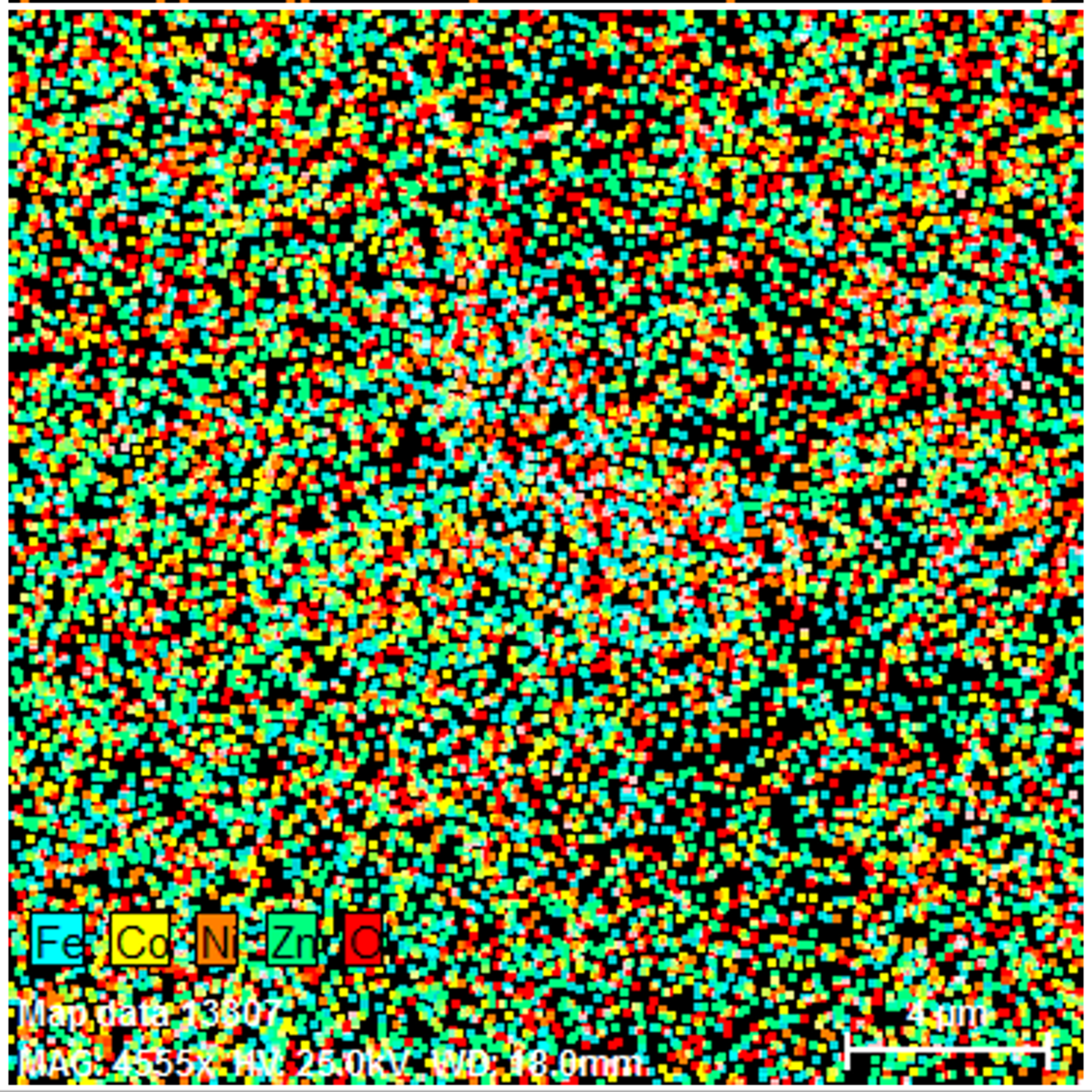

Supplement: Supplementary file 1 [file DataSheet2.zip › S2/S2,G.tif]
