# Supplementary material for: Solar light induced photocatalytic degradation of tetracycline in the presence of ZnO/NiFe2O4/Co3O4 as a new and highly efficient magnetically separable photocatalyst
Source: Front Chem. 2022 Oct 13;10:1013349. doi: 10.3389/fchem.2022.1013349 (PMC9606596; doi:10.3389/fchem.2022.1013349)
Supplement: Supplementary file 2 [file DataSheet1.docx]

Supplementary Material

# Supplementary Figures


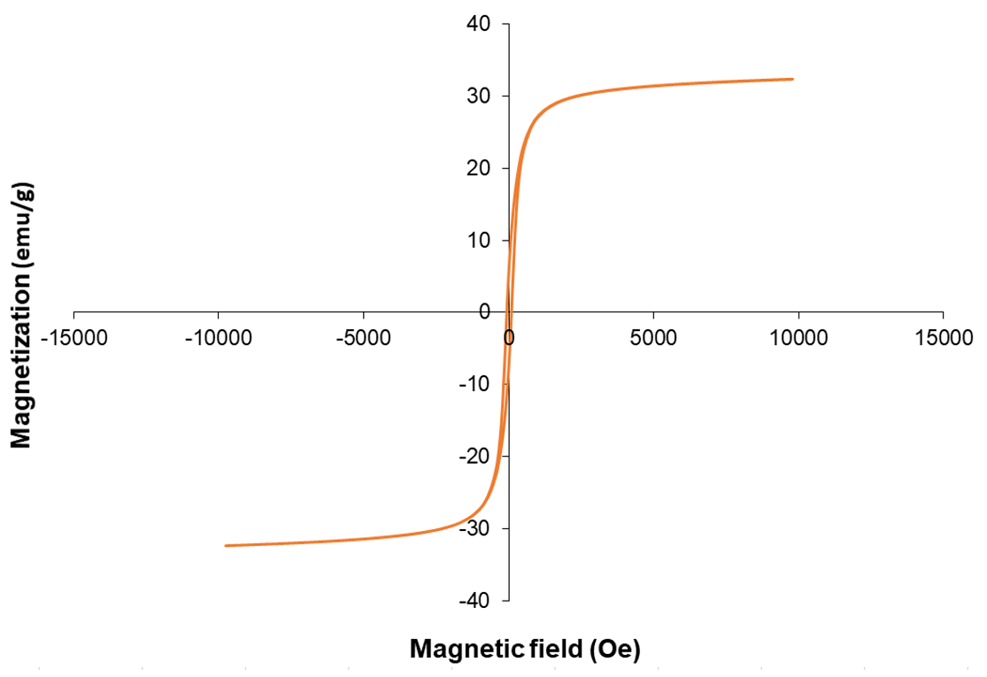


**Figure S1.** Magnetization curve of ZnO/NiFe_2_O_4_/Co_3_O_4_.

**(A)**


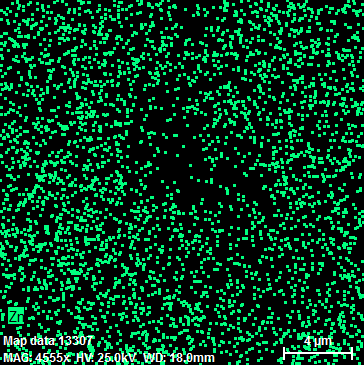

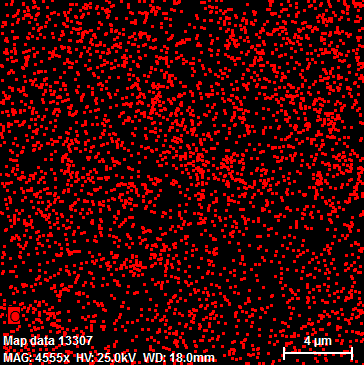

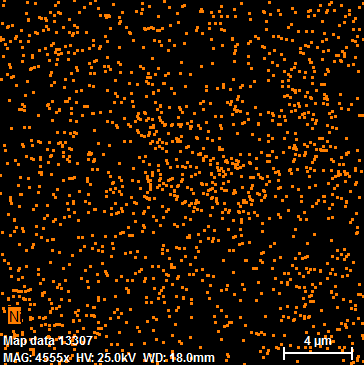

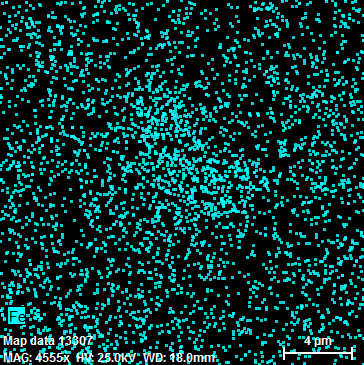

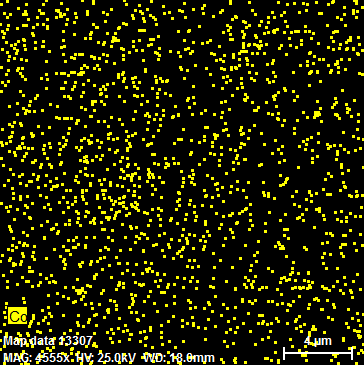

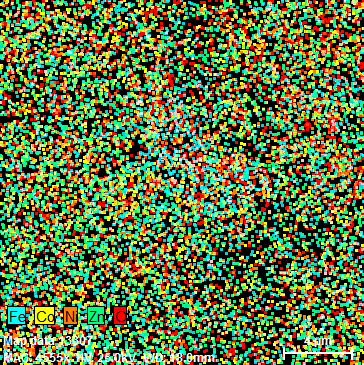


**(B)**

**(C)**

**(D)**

**(E)**

**(F)**

**(G)**

**Figure S2.** (**A**) EDS analysis of the ZnO/NiFe_2_O_4_/Co_3_O_4_ and mapping images of (**B**) zinc (green), (**C**) oxygen (red), (**D**) nickel (orange), (**E**) iron (blue), (**F**) cobalt (yellow) and (**G**) the overlapping of Zn, O, Ni, Fe and Co in ZnO/NiFe_2_O_4_/Co_3_O_4_.


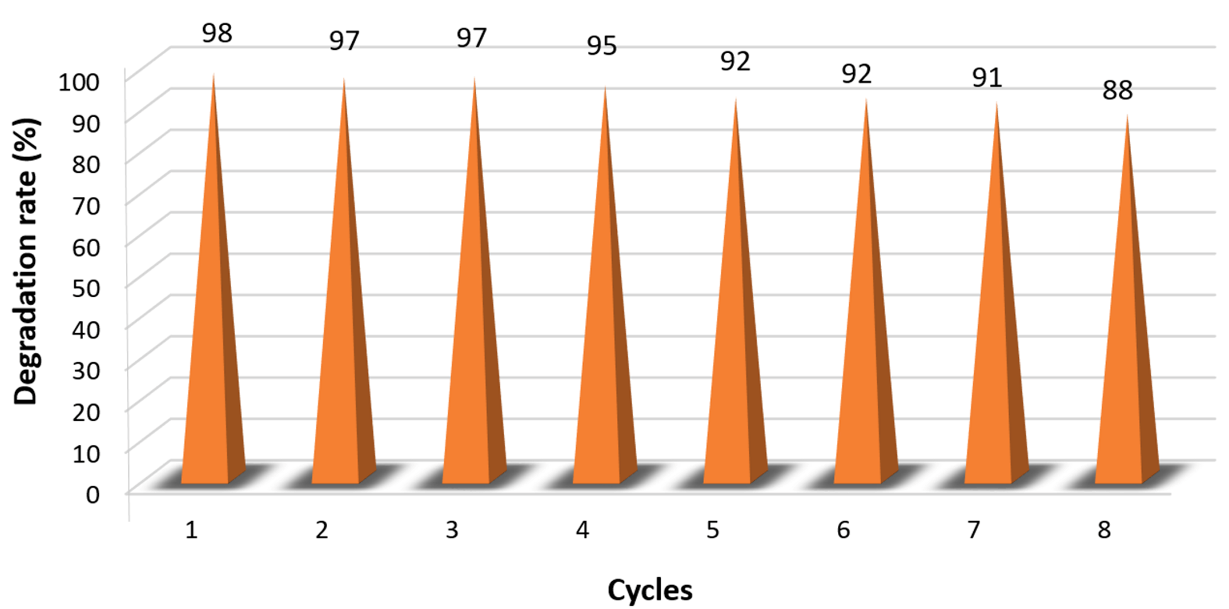


**Figure** **S3.** Recycling exploration of ZnO/NiFe_2_O_4_/Co_3_O_4_ nanocomposite in the photocatalytic degradation of TC.


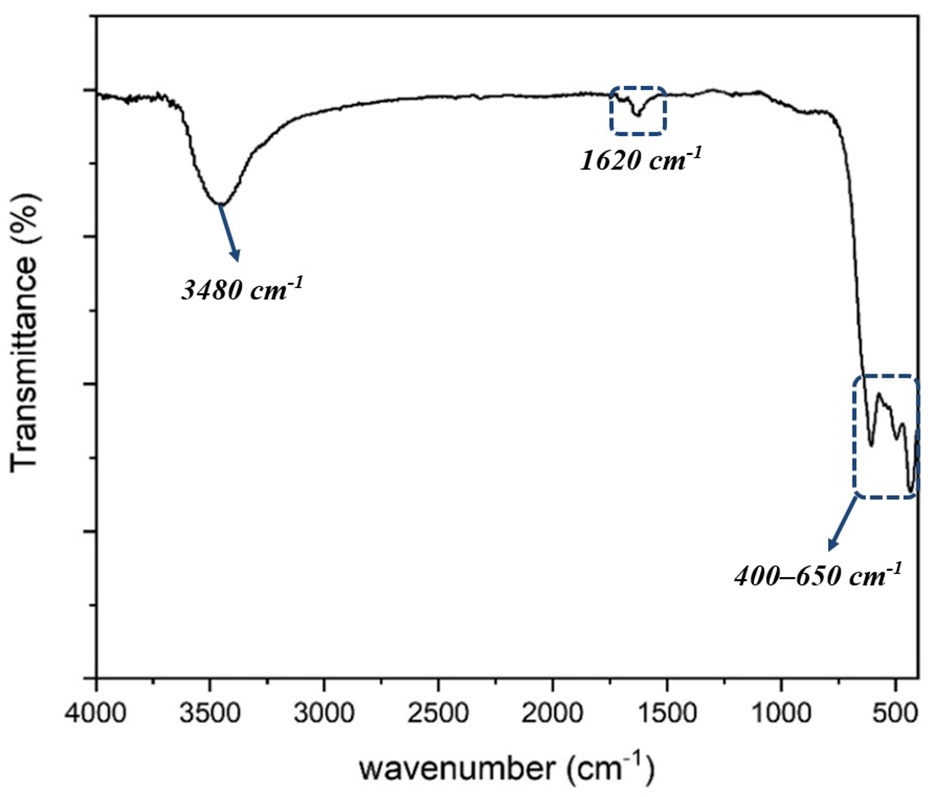


**Figure S4.** FT-IR of ZnO/NiFe_2_O_4_/Co_3_O_4_ after eight times reused.


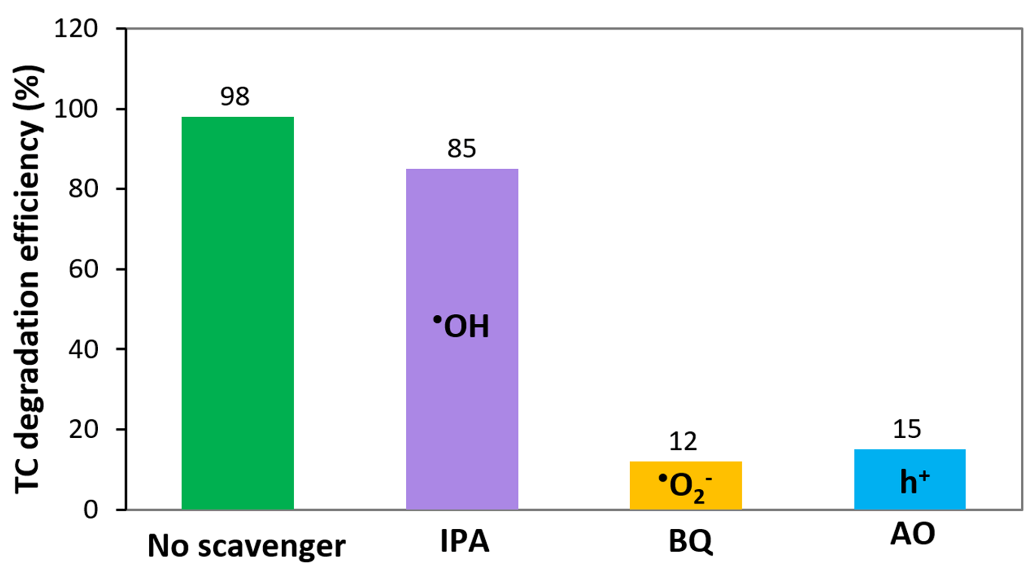


**Figure S5.** The progress of TC photocatalytic degradation using ZnO/NiFe_2_O_4_/Co_3_O_4_ in the presence of different scavengers in optimized conditions.
